# DCAF13 Safeguards Hematopoietic Stem Cells via RRS1‐Regulated Ribosome Biogenesis

**DOI:** 10.1002/advs.202513777

**Published:** 2026-03-06

**Authors:** Mengke Li, Yuxin Wu, Shuai Zhou, Peipei Tang, Jiaming Wei, Ling Liu, Zhenyi Wang, Deyang Shi, Shengnan Yuan, Qingyu Zhang, Shihui Wang, Qing Zhang, Huan Zhao, Rui Zhang, Yingying Wang, Shiwei Yang, Xiangli Chen, Xiaojing Shi, Guangzhi Liu, Yuwei Zhang, Jinming Li, Xu Dong Zhang, Rick F Thorne, Dongping Wei, Zumin Zhu, Song Chen

**Affiliations:** ^1^ Institute of Hematology Henan Key Laboratory of Stem Cell Differentiation and Modification Henan Provincial People's Hospital Zhengzhou University People's Hospital Zhengzhou China; ^2^ Translational Research Institute Henan International Join Laboratory of Non‐coding RNA and Metabolism in Cancer,Henan Provincial People's Hospital,Academy of Medical Sciences Zhengzhou University Zhengzhou Henan China; ^3^ Jiangsu Province Engineering Research Center of Precise Prevention in the Digestive and Reproductive System Cancers Institute of Medicinal Biotechnology Jiangsu College of Nursing Huai'an Jiangsu China; ^4^ Medical Research Center The First Affiliated Hospital of Wenzhou Medical University Wenzhou Zhejiang China; ^5^ School of Medical Technology Xuzhou Medical University Xuzhou Jiangsu China; ^6^ Laboratory Animal Center Academy of Medical Sciences Zhengzhou University Zhengzhou Henan China

**Keywords:** DCAF13, HSC, P53, ribosome biogenesis, RRS1

## Abstract

Hematopoietic stem cells (HSCs) sustain lifelong blood production by balancing self‐renewal and differentiation. The mechanisms regulating HSC homeostasis, particularly those involving ribosome biogenesis, remain incompletely understood. Here, we identify DCAF13 as a critical regulator of HSC maintenance by stabilizing RRS1, a key factor in ribosome biogenesis. Conditional deletion of *Dcaf13* in murine hematopoietic cells results in severe pancytopenia, rapid mortality, and complete HSC depletion in both fetal and adult hematopoietic compartments. We show that DCAF13 deficiency disrupts ribosome assembly and protein synthesis, selectively affecting the translation of mRNAs from genes involved in myeloid differentiation, chromatin remodeling, and erythroid homeostasis. DCAF13 directly binds RRS1 and catalyzes its K27‐linked polyubiquitination, a non‐degradative post‐translational modification that enhances RRS1 protein stability.While *Dcaf13* deletion activates the p53 pathway, *Trp53* ablation only partially restores HSC numbers and cell cycle progression, and does not prevent apoptosis and hematopoietic failure, indicating the involvement of both p53‐dependent and p53‐independent mechanisms. These findings establish a DCAF13‐RRS1 axis essential for HSC function, in which DCAF13 acts as an essential regulator of ribosome biogenesis. This work provides molecular insights into the pathogenesis of hematopoietic disorders and ribosomopathies.

## Graphical abstract

This study establishes DCAF13 as an essential regulator for hematopoietic stem cell (HSC) function. Its deletion in mice causes lethal pancytopenia and HSC depletion. Mechanistically, DCAF13 interacts with RRS1 and mediates its non‐degradative K27‐linked ubiquitination, thereby stabilizing RRS1 to maintain ribosome biogenesis and protein translation, which defines a critical DCAF13‐RRS1 axis for hematopoietic disorders and ribosomopathies.

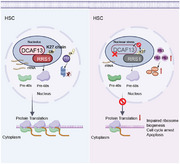

## 1 Introduction

Hematopoietic stem cells (HSCs) sustain lifelong blood production through a tightly regulated balance of quiescence, proliferation, and differentiation [1, 2]. Disruptions in these mechanisms can lead to hematopoietic disorders such as bone marrow failure syndromes, myelodysplastic syndromes, and leukemia [3], highlighting the need to elucidate molecular regulators of HSC homeostasis.

Ribosome biogenesis, the process of assembling ribosomes and regulating protein translation, is increasingly recognized as a key determinant of stem cell fate and function [4, 5, 6, 7]. Far from being a constitutive housekeeping function, this process is tightly regulated to maintain stem cell homeostasis. This is underscored by bone marrow failure syndromes, such as Diamond‐Blackfan anemia and Shwachman‐Diamond syndrome, which arise from mutations in ribosomal components [8, 9, 10, 11]. These clinical manifestations highlight the exquisite sensitivity of hematopoietic stem cells to perturbations in ribosome production.

DDB1‐ and CUL4‐associated factor 13 (DCAF13) functions as a substrate receptor for the CUL4‐DDB1 E3 ubiquitin ligase complex and localizes to the nucleolus, suggesting a potential role in ribosome biogenesis [12, 13]. As part of the CUL4‐DDB1 complex that regulates diverse cellular processes, including DNA repair, cell cycle control, and protein turnover [14], DCAF13 confers substrate specificity for targeted protein degradation [15]. While nucleolar stress from impaired ribosome biogenesis typically activates p53‐dependent pathways affecting HSC quiescence and stress responses [16, 17, 18, 19], emerging evidence reveals that additional p53‐independent mechanisms exist in stem cells and cancer cells [20, 21, 22, 23, 24]. Despite these advances, how DCAF13 specifically connects ribosome biogenesis to HSC regulation remains poorly understood.

Here, using a genetic knockout approach, we demonstrate that Dcaf13 deficiency leads to severe hematopoietic failure, including HSC depletion and impaired reconstitution capacity. Mechanistically, Dcaf13‐deficient HSCs exhibit abnormal ribosome assembly, reduced protein synthesis rates, and activation of p53 signaling. However, while *Trp53* ablation partially restored phenotypic HSC numbers and cell cycle distribution, it failed to prevent hematopoietic failure. Notably, we uncovered a novel interaction between DCAF13 and RRS1, which is crucial for ribosome assembly and protein synthesis in HSCs.

Our findings establish DCAF13 as a key regulator of HSC maintenance and provide new insights into the intricate relationship between nucleolar function, ribosome biogenesis, and HSC fate.

## 2 Results

### 2.1 Conditional Loss of *Dcaf13* Leads to Perinatal Death and Exhaustion of Fetal Liver HSCs

**FIGURE 1:**
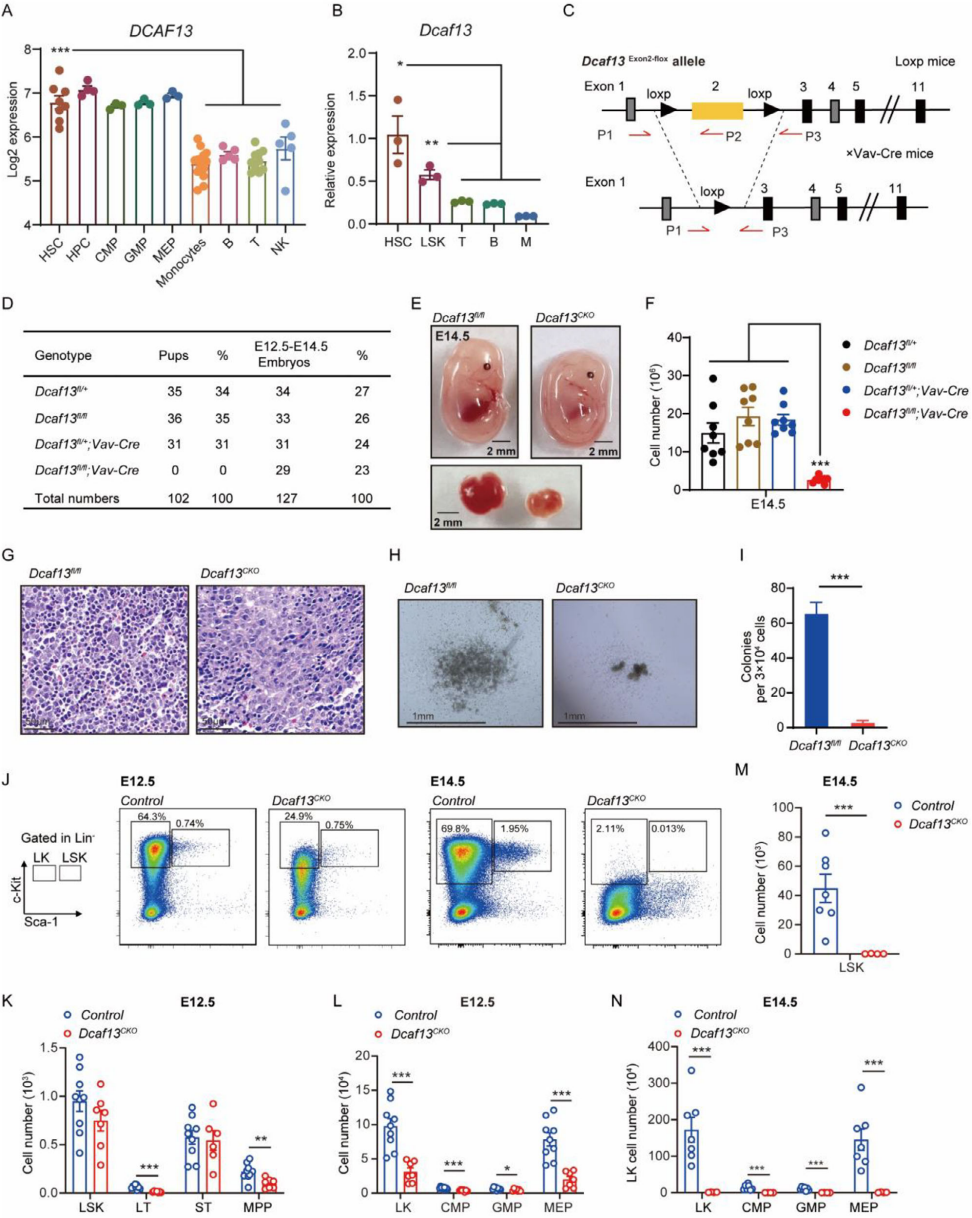
Conditional loss of *Dcaf13* leads to perinatal death and exhaustion of fetal liver HSCs. (A) The expression level of *DCAF13* in normal human blood cells (data obtained from BloodSpot); one‐way ANOVA; ***p < 0.001. (B) The expression pattern of *Dcaf13* in murine hematopoietic stem cells (HSC), LSK cells, T cells, B cells and mature myeloid cells were analyzed. mRNA levels were normalized to β‐actin expression; n = 3. (C) The targeting strategy to generate the *Dcaf13*‐loxp and *Dcaf13^fl/fl^;Vav‐iCre* mice (abbreviated as *Dcaf13^CKO^*) was shown. Arrows indicate the location of genotyping primers. (D) The number and percentage of each genotype in the newborn pups and E12.5‐E14.5 embryos were assessed by mating *Dcaf13^fl/fl^* mice with *Dcaf13^fl/+^;Vav‐iCre* mice. (E) Representative images of *Dcaf13^fl/fl^*, *Dcaf13^fl/fl^;Vav‐iCre* (abbreviated as *Dcaf13^CKO^*) embryos and fetal livers at E14.5. (F) The cell numbers of E14.5 fetal livers (abbreviated as FL). (G) Representative H&E staining of fetal liver sections from E14.5 embryos. Scale bars: 50 µm. (H) Representative images of colonies after 2‐week culture in methylcellulose medium. Scale bar: 1 mm. (I) Colony number analysis of E14.5 fetal liver cells, n = 3. (J) Representative FACS analysis of fetal liver cells from control and *Dcaf13^CKO^* embryos at E12.5 and E14.5. *Dcaf13^fl/fl^* and *Dcaf13^fl/+^* were assigned control group. The analyzed population include LSK^+^s (Lin^−^Sca1^+^c‐Kit^+^), LT‐HSC (Lin^−^Sca1^+^c‐Kit^+^CD34^−^Flt3^low^), ST‐HSC (Lin^−^Sca1^+^c‐Kit^+^CD34^+^Flt3^low^), MPP (Lin^−^Sca1^+^c‐Kit^+^CD34^+^Flt3^+^), LK (Lin^−^c‐Kit^+^Sca‐1^−^), CMP (Lin^−^c‐Kit^+^Sca1^−^CD34^+^CD16/32^low^), GMP (Lin^−^c‐Kit^+^Sca1^−^CD34^+^CD16/32^high^) and MEP (Lin^−^c‐Kit^+^Sca1^−^CD34^−^CD16/32^low^). (K, L) The absolute number of LSK, LT‐HSC, ST‐HSC, MPP, LK, CMP, GMP, and MEP in control and *Dcaf13^CKO^* E12.5 fetal livers (n  =  9 in control and 7 in *Dcaf13^CKO^*). (M, N) The absolute number of LSK, LK, CMP, GMP, MEP in control and *Dcaf13^CKO^* E14.5 fetal livers (control, n = 7; *Dcaf13^CKO^, n = 4*). Data are presented as Mean ± SEM; *p < 0.05, **p < 0.01, ***p < 0.001, Student's t‐test.

Previous studies have shown that germline deletion of Dcaf13 results in early embryonic lethality in mice [25], while oocyte‐specific deletion causes female sterility [26]. However, the role of DCAF13 in other tissues and organ systems remains unexplored. To investigate its potential role in hematopoiesis, we performed a bioinformatic analysis of the human hematopoietic differentiation cascade using curated bulk RNA‐seq datasets (BloodSpot) and single‐cell RNA‐seq data (ABC portal) [27]. DCAF13 expression was markedly higher in hematopoietic stem and progenitor cells (HSPCs) compared with mature blood cell populations (Figure 1A; Figure S1A). Notably, DCAF13 expression was significantly reduced in HSCs from patients with Diamond–Blackfan anemia (Figure S1B), providing a possible link between DCAF13 dysfunction and human ribosomopathies. In mice, qPCR analysis of hematopoietic subsets revealed elevated Dcaf13 expression in HSCs and LSK cells relative to mature T, B, and myeloid cells (Figure  1B). Western blot analyses of murine Lin^−^c‐Kit^+^ cells and human CD34^+^ cells further confirmed higher DCAF13 protein abundance in HSPCs (Figure S1C). Collectively, the selective enrichment of DCAF13 in HSPCs, together with its downregulation in DBA, implicates DCAF13 as a potential regulator of both normal hematopoiesis and hematopoietic disorder pathogenesis.

In light of the preceding findings, we sought to investigate the hematopoietic role of *Dcaf13* using mouse genetic models. We first attempted to generate conditional knockout mice by crossing floxed *Dcaf13* (*Dcaf13^fl/fl^*) mice with *Vav‐iCre* mice to generate *Dcaf13^fl/fl^;Vav‐iCre* mice (hereafter referred to as *Dcaf13^CKO^*) (Figure 1C; Figure S1D). However, the *Dcaf13^CKO^* genotype was absent in newborn mice (Figure 1D) although the expected Mendelian ratios of *Dcaf13^CKO^* embryos and littermate controls (*Dcaf13^fl/+^*, *Dcaf13^fl/fl^*, *Dcaf13^fl/+^;Vav‐iCre*) were observed at embryonic days E12.5‐E14.5 (Figure 1D), suggestive of late embryonic lethality.

We next examined fetal livers, noting that the active and terminal stages of fetal liver hematopoiesis in mice occur between E14.5‐E18.5 [1, 28, 29, 30]. *Dcaf13^CKO^* embryos at E12.5‐E14.5 displayed paler livers compared to other genotypes with significant reductions occurring in liver size and cellularity (Figure 1E,F; Figure S1E). Similarly, *Dcaf13^CKO^* fetal livers appeared paler and smaller at E16.5 and E18.5 (Figure S1F). Histopathological assessment with HE staining revealed noticeable reductions in hematopoietic cells within *Dcaf13^CKO^* fetal livers (Figure 1G), suggesting that ablation of *Dcaf13* in HSCs leads to perinatal lethality associated with impaired fetal hematopoiesis.

To further characterize the hematopoietic defect in *Dcaf13^CKO^* mice, we performed colony‐forming unit (CFU) assays and immunophenotyping of hematopoietic stem and progenitor cell subsets (HSPCs). Notably, the number of colonies derived from E14.5 *Dcaf13^CKO^* fetal livers was significantly lower than from E14.5 *Dcaf13^fl/fl^* littermates (Figure 1H,I), suggesting that *Dcaf13* deletion impairs fetal HSPC development. Further examination of HSPCs using flow cytometry revealed no significant changes in LSK and ST‐HSC cell numbers in E12.5 fetal livers (Figure 1J,K), although the numbers of LT‐HSC, MPP, LK, CMP, GMP, and MEP cells were decreased in *Dcaf13^CKO^* embryos (Figure 1L; Figure S1G). Subsequent analyses at E14.5 revealed dramatic reductions in the number of LSK, LK, CMP, GMP, and MEP cells in *Dcaf13^CKO^* fetal livers (Figure 1J,M,N). Additional interrogation of c‐Kit expression showed its expression was patently decreased in E12.5 and E14.5 fetal liver cells from *Dcaf13^CKO^* embryos (Figure S1H). Accompanying analysis of erythroid maturation using the CD71 and Ter119 surface markers indicates compromised erythroid maturation in E12.5 and E14.5 *Dcaf13^CKO^* fetal livers, as shown by absolute reductions in the number of erythroid populations (Figure S1I–K). Taken together, these results suggest a crucial role for *Dcaf13* in fetal erythropoiesis and demonstrate that its loss in the hematopoietic lineage leads to fetal HSPC depletion.

### 2.2 DCAF13 Knockout Impairs the Reconstitution Capacity of Fetal Liver HSCs

**FIGURE 2:**
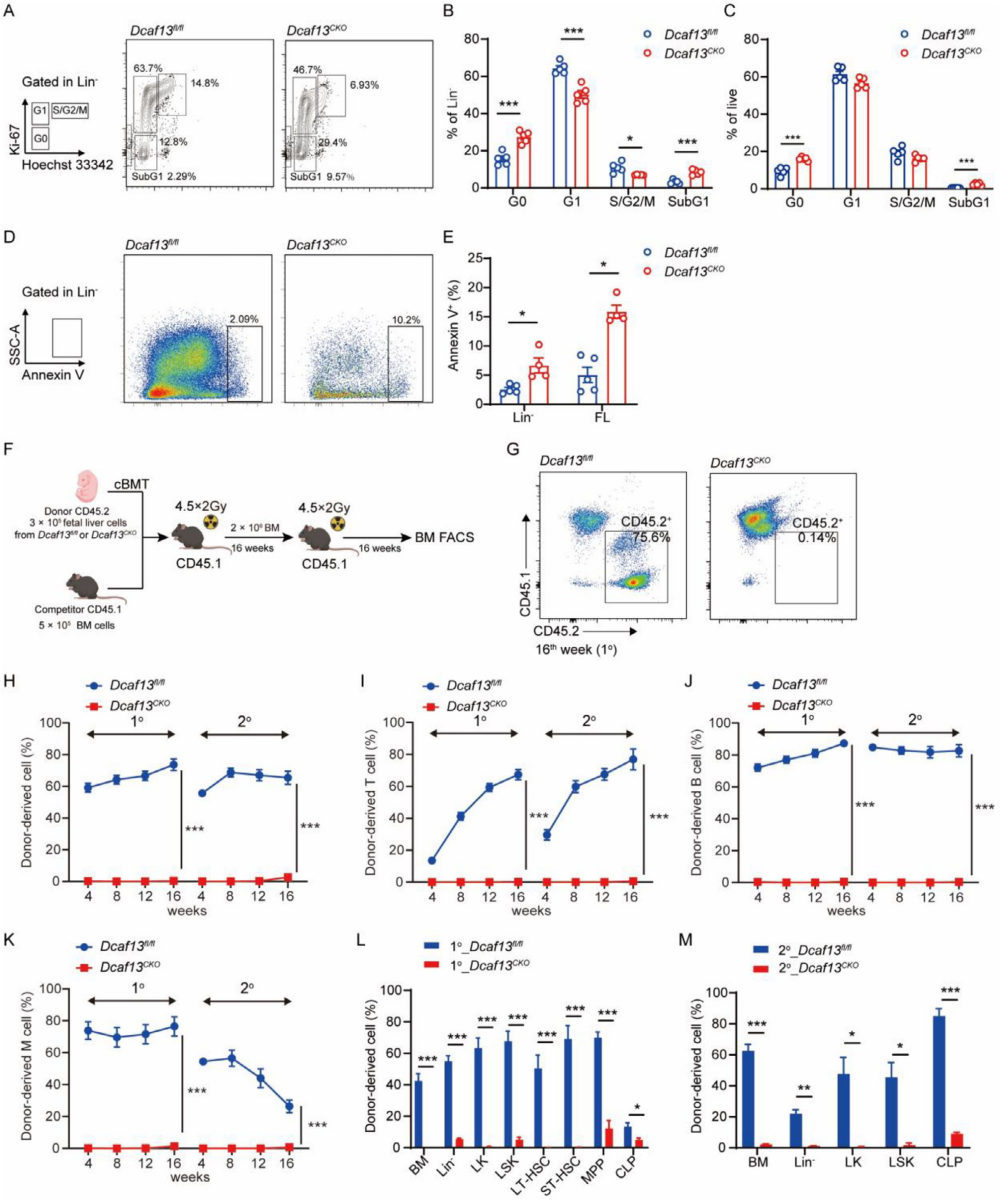
DCAF13 deletion impairs the reconstitution capacity of fetal liver HSCs. (A) Representative cell cycle analysis of Lin^−^ cells from E14.5 *Dcaf13^fl/fl^* and *Dcaf13^CKO^* fetal livers using Ki67 and Hoechst 33342 staining. (B, C) The percentage of G0, G1, S/G2/M, and SubG1 phases in Lin^−^ and total cells are shown for E14.5 fetal livers; n = 5. (D, E) Apoptosis analysis of E14.5 fetal livers and Lin^−^ cells from *Dcaf13^fl/fl^* and *Dcaf13^CKO^* embryos (n  =  5 in *Dcaf13^fl/fl^* and n = 4 in *Dcaf13^CKO^*). (F) Strategy for serial competitive repopulation assays, cBMT: competitive bone marrow transplantation. (G) Flow cytometry analysis of the percentage of donor‐derived cells (CD45.2^+^) in the PB of CD45.1^+^ recipient mice at 16 weeks post‐transplantation. (H–K) Quantification of donor‐derived (CD45.2) cells (H), donor‐derived T (CD3^+^) cells (I) and B (B220^+^) cells (J), and donor‐derived myeloid cells (K) in the PB at the indicated time points in primary (n = 8) and secondary (n = 6) recipients. (L, M) Donor contribution of indicated cell populations in BM cells of primary and secondary recipient mice at 16 weeks post‐transplantation; n = 5. Data are presented as Mean ± SEM; *p < 0.05, **p < 0.01, ***p < 0.001, Student's t‐test.

To investigate the basis for the depletion of HSPCs in *Dcaf13^CKO^* embryos, we first analyzed the cell cycle using Ki67 and Hoechst 33342 staining. The analysis of Lin^−^ cells revealed increased proportions of G0 phase cells with accompanying decreases in the G1 and S/G2/M phases from *Dcaf13^CKO^* fetal livers. Furthermore, increased numbers of total and Lin^−^ *Dcaf13^CKO^* liver cells accumulated in the subG1 gate, indicative of DNA fragmentation associated with apoptosis (Figure 2A–C). Consistently, increased apoptosis rates in *Dcaf13^CKO^* liver cells were verified using Annexin V staining (Figure 2D,E; Figure S2A). Additional immunohistochemical (IHC) analyses revealed significantly lower staining for the Ki67 proliferative marker in E14.5 *Dcaf13^CKO^* fetal livers (Figure S2B). Together, these findings demonstrate that the depletion of HSPCs in *Dcaf13*‐knockout fetal livers was due to impaired cell cycle progression and increased apoptosis.

Lastly, to assess the impact of *Dcaf13* deficiency on the long‐term hematopoietic reconstitution capacity of HSCs, we performed competitive serial transplantation assays. Lethally irradiated CD45.1 mice were reconstituted with mixtures of CD45.2 fetal liver cells from E14.5 embryos and competitor CD45.1 BM cells (Figure 2F). Nevertheless, flow cytometric analyses of peripheral blood (PB) and bone marrow (BM) failed to detect CD45.2^+^ donor‐derived cells arising from *Dcaf13^CKO^* fetal livers in both primary and secondary transplantation assays (Figure 2G,H). Mature donor‐derived T cells (CD3^+^), B cells (B220^+^), and myeloid cells (CD11b^+^ Gr1^+^) were also similarly undetectable at 4, 8, 12, and 16 weeks post‐transplantation (Figure 2I–K). Targeted analyses of *Dcaf13^CKO^* HSPCs in recipient BM further showed significantly reduced chimerism at 16 weeks post‐primary and secondary transplantation (Figure 2L,M). Collectively, these results indicate that *Vav‐iCre*‐mediated *Dcaf13* deletion impairs HSPC function, leading to rapid exhaustion of the HSC pool and embryonic lethality.

### 2.3 DCAF13 is Essential for the Maintenance of Adult HSC Pools

**FIGURE 3:**
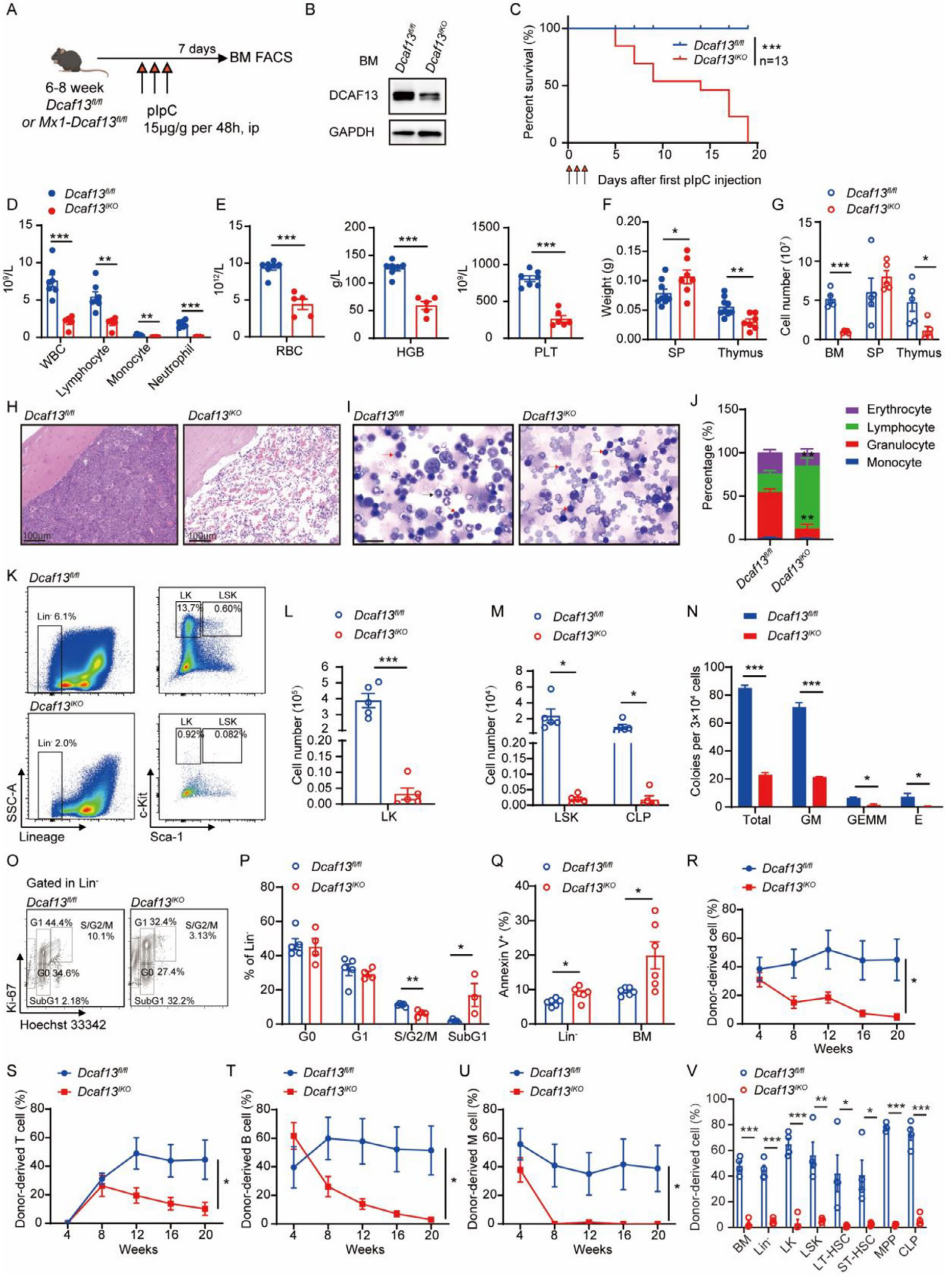
DCAF13 is essential for the maintenance of the adult HSC pool. (A) Schematic diagram of Mx1‐Cre mediated *Dcaf13* knockout in adult hematopoiesis induced by intraperitoneally (IP) injection of poly(I:C) (pIpC) at 15 mg/kg every other day for three consecutive doses. (B) Western blot analysis of DCAF13 level in *Dcaf13^fl/fl^;Mx1‐Cre* BM cells after pIpC treatment (abbreviated as *Dcaf13^IKO^*). (C) Kaplan–Meier survival curve of mice following pIpC‐induced *Dcaf13* knockout (n = 13). Red arrows indicate pIpC injection time points. (D, E) Complete blood count analysis of *Dcaf13* knockout and control mice after pIpC injections. WBC (white blood cells); RBC (red blood cells); HGB (hemoglobin); PLT (platelets). (n  =  7 in *Dcaf13^fl/fl^* and n = 5 in *Dcaf13^IKO^*). (F) The weight of spleen (SP) and thymus in *Dcaf13* knockout and control mice after pIpC injections; n = 7–9. (G) The cell numbers of BM, SP, and thymus. Femur, tibia, and fibula were calculated for BM cellularity; n = 5. (H) H&E staining of mouse femur sections seven days after three pIpC injections. (I, J) Wright–Giemsa staining of the cytospin of BM cells. The histogram shows the relative proportions of erythroid cells, lymphocytes, granulocytes, and monocytes within BM, n = 3; black arrows show granulocytes (*Dcaf13^fl/fl^*) and red arrows show lymphocytes (*Dcaf13^IKO^*); (K) FACS analysis of LSK cells in the BM of *Dcaf13^fl/fl^* and *Dcaf13^IKO^* mice after pIpC injections. (L, M) The cell number of LKs, LSKs, and CLPs in the BM of *Dcaf13^fl/fl^* and *Dcaf13^IKO^* mice; n = 5. (N) Colony number analysis of the BM cells between *Dcaf13^fl/fl^* and *Dcaf13^IKO^* mice; n = 3. (O) Representative FACS profiles of Ki67 staining of Lin^−^ cells in the BM of *Dcaf13^fl/fl^* and *Dcaf13^IKO^* mice. (P) The frequencies of G0, G1, and S/G2/M phases are shown; n = 4. (Q) Apoptosis analysis of BM cells in *Dcaf13^fl/fl^* and *Dcaf13^IKO^* mice; n = 6. (R–U) Quantification of donor‐derived (CD45.2^+^) cells in the PB of recipient mice at the indicated time points; n = 5. (V) Donor contribution of indicated cell populations in the BM of recipient mice 20 weeks post‐transplantation; n = 4. Data are presented as Mean ± SEM; *p < 0.05, **p < 0.01, ***p < 0.001, Student's t‐test.

Given the preceding findings of embryonic lethality, we generated *Dcaf13^fl/fl^;Mx1‐Cre* mice to investigate the function of *Dcaf13* in adult HSCs (hereafter termed *Dcaf13^IKO^*; Figure S2C). Following pIpC treatment, we confirmed efficient ablation of Dcaf13 in bone marrow after 7 days (Figure 3A,B). Nonetheless, all *Dcaf13* knockout mice died within 20 days following the initial pIpC injection (Figure 3C) with blood count analyses showing reductions in white blood cells (WBC), lymphocytes, monocytes, and neutrophils relative to control littermates (Figure 3D). *Dcaf13^IKO^* mice also showed severely reduced numbers of RBCs, HGB, and platelets (Figure 3E). Necroscopic examinations showed that *Dcaf13^IKO^* mice exhibited splenomegaly and thymic atrophy characterized by prominent splenic nodules and decreased thymus weights (Figure 3F; Figure S2D). Consistently, the cellularity of BM and thymus was decreased in *Dcaf13^IKO^* mice (Figure 3G), while accompanying histopathological examination of femurs and sternum confirmed significant decreases in *Dcaf13^IKO^* BM cellularity (Figure 3H; Figure S2E). However, morphologic counting of BM and PB showed an elevated proportion of lymphocytes in *Dcaf13^IKO^* mice using cytospin preparations (Figure 3I,J; FigureS2F).

To better dissect the hematopoietic changes in *Dcaf13^IKO^* mice, we undertook immunophenotyping analyses. Seven days after pIpC treatment, we observed decreases in both myeloid and B220^+^ cells in *Dcaf13^IKO^* BM relative to controls (Figure S3A). In contrast, T cell percentages in PB were elevated in *Dcaf13^IKO^* mice (Figure S3B), while the numbers of thymic T cells were substantially diminished (Figure S3C), likely accounting for the lymphocytosis in PB. We also observed a dramatic decrease in R1‐R4 erythrocyte subpopulations in *Dcaf13^IKO^* mice, although with no detectable change in mature erythrocytes (Figure S3D,E), the latter suggesting compensatory erythropoiesis in response to hypoplasia of other hematopoietic lineages.

Parallel examination of HSPCs in *Dcaf13^IKO^* mice recorded profound depletions in LK, LSK, and CLP subsets compared with controls (Figure 3K–M). Functional evaluation using CFU assays also demonstrated that BM from *Dcaf13^IKO^* mice possessed reduced clonogenic capacity (Figure 3N; Figure S3F). Accompanying cell cycle analysis also revealed decreased proportions of S/G2/M phase in *Dcaf13^IKO^* Lin^−^ cells with concomitant subG1‐phase accumulation (Figure 3O,P). Consistently, increased apoptotic levels were detected in BM Lin^−^ cells from *Dcaf13^IKO^* mice using Annexin V staining (Figure 3Q; Figure S3G,H), together suggesting hematopoietic defects in *Dcaf13^IKO^* HSPCs.

To verify this, we undertook competitive transplantation assays using the CD45 alloantigen system. We mixed 5 × 10^5^ BM cells (CD45.2) from untreated *Dcaf13^fl/fl^* or *Dcaf13^fl/fl^;Mx1‐Cre* mice with an equal number of competitor BM cells (CD45.1) and transplanted these into lethally irradiated CD45.1 recipients. Peripheral blood chimerism was assessed by flow cytometry at 4 weeks post‐transplantation to confirm engraftment with subsequent three injections of pIpC to induce Dcaf13 deletion (Figure S3I). Subsequent PB analyses showed progressive decreases in the chimerism of mature blood cell lineages in *Dcaf13^fl/fl^;Mx1‐Cre* recipient mice (Figure 3R–U) with assessment of BM also detecting significantly reduced chimerism in *Dcaf13* knockout HSPCs (Figure 3V), consistent with a profound competitive fitness deficiency in Dcaf13‐deficient HSPCs.

To definitively demonstrate these findings resulted from cell‐intrinsic defects in HSPCs, we transplanted 1000 LSK cells (CD45.2) from *Dcaf13^fl/fl^* or *Dcaf13^fl/fl^;Mx1‐Cre* mice, along with competitor bone marrow cells (CD45.1), into lethally irradiated CD45.1 recipients. After confirming engraftment and inducing *Dcaf13* deletion with pIpC (Figure S3J–K), we again detected the progressive decline in the chimerism of donor‐derived mature blood cells from *Dcaf13^fl/fl^;Mx1‐Cre* mice in peripheral blood (Figure S3L,M). This was coupled with a significant reduction in chimerism of Dcaf13‐deficient HSPCs in the bone marrow (Figure S3N). Thus, phenocopying the effects of Dcaf13 on fetal hematopoiesis, deletion of *Dcaf13* using Mx1‐Cre profoundly depletes adult HSC pools and impairs hematopoietic function.

### 2.4 *Dcaf13*‐Deficient HSCs Exhibit Activation of P53 Signaling and Impaired Ribosome Biogenesis

**FIGURE 4:**
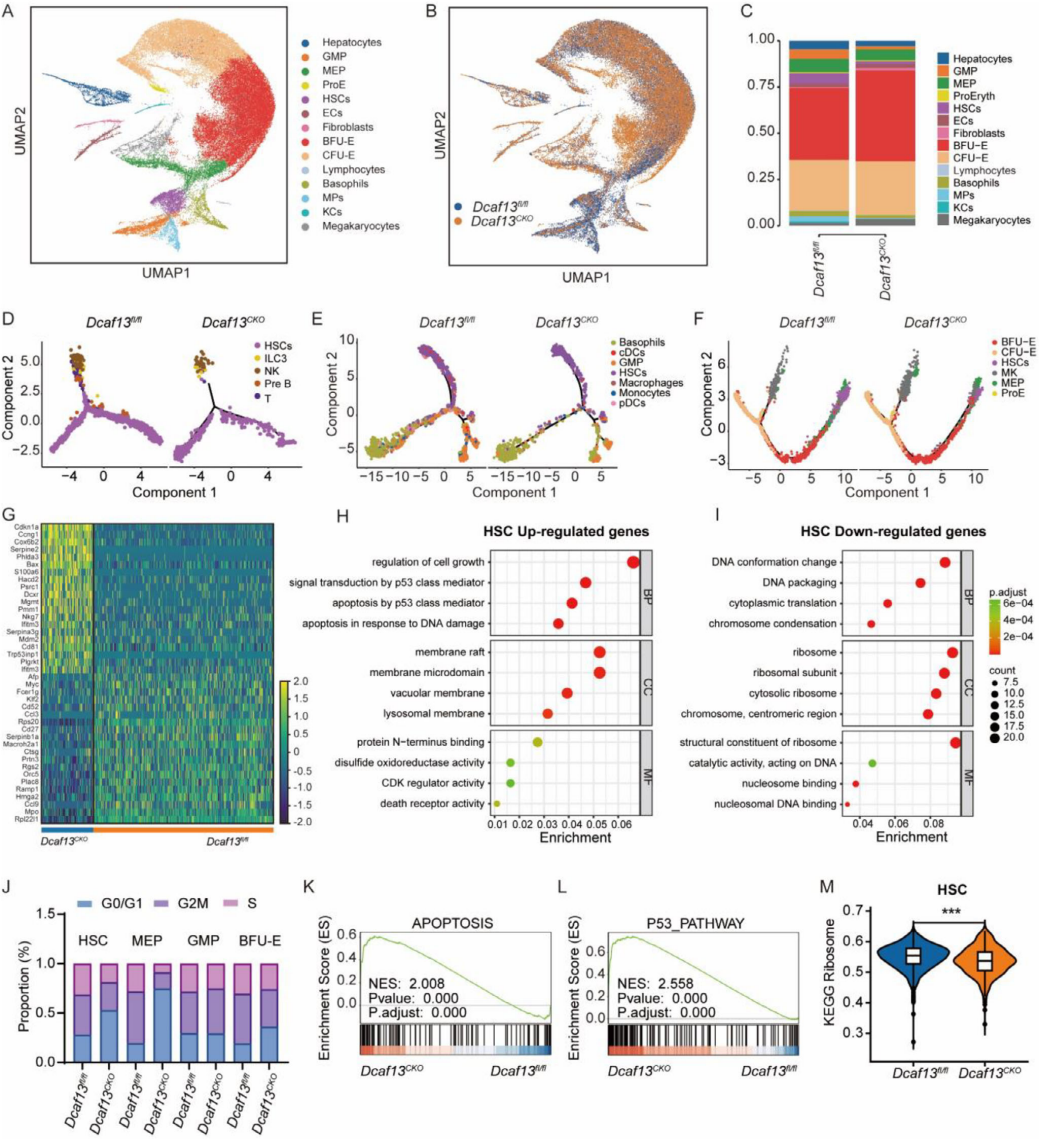
*Dcaf13*‐deficient HSCs exhibit activation of the p53 signaling pathway and impaired ribosome biogenesis. (A) UMAP visualization of single‐cell transcriptomes of identified clusters using Seurat, 65 478 cells were processed for scRNA‐seq and analysis. (B) UMAP visualization of E14.5 Lin^−^ cells from *Dcaf13^fl/fl^* and *Dcaf13^CKO^* embryos. (C) Histograms showing the compositions of transcriptome‐defined 14 clusters in the two groups. (D–F) Pseudotime analysis following sub‐clustering revealed HSC differentiation into three lineages: the lymphoid branch, comprising T cells, pre‐B cells, natural killer cells (NK), and innate lymphoid cells type 3 (ILC3); the myeloid branch, including GMPs, macrophages, monocytes, plasmacytoid dendritic cells (pDC), and conventional dendritic cells (cDC); and the erythroid/megakaryocytic branch, encompassing MEPs, BFU‐Es, CFU‐Es, pro‐erythroblasts (ProE), and megakaryocytes. (G) Heatmap of differentially expressed genes (DEGs) in *Dcaf13^fl/fl^* and *Dcaf13^CKO^* HSC. (H) GO enrichment pathway of up‐regulated genes in *Dcaf13^CKO^* HSC compared with *Dcaf13^fl/fl^* HSC. (I) Enrichment analysis of down‐regulated genes in *Dcaf13^CKO^* HSC compared to *Dcaf13^fl/fl^* HSCs. (J) Proportions of HSCs, MEPs, GMPs, and BFU‐E cells identified with Seurat in each of the cell cycle stages between the two groups. (K, L) Gene set enrichment analysis (GSEA) of apoptosis and P53 pathway in *Dcaf13^fl/fl^* and *Dcaf13^CKO^* HSC. (M) Violin plot showing differences in ribosome biogenesis between *Dcaf13^fl/fl^* and *Dcaf13^CKO^* HSCs.

To unravel the molecular basis underlying *Dcaf13* regulation of HSC homeostasis, we performed single‐cell RNA sequencing (scRNA‐seq) comparing Lin^−^ cells isolated from E14.5 *Dcaf13^fl/fl^* and *Dcaf13^CKO^* embryos. The processed scRNA‐seq library yielded a total of 65 478 cells, grouped into 14 clusters based on transcriptional signatures. Among these, ten clusters corresponded to hematopoietic cell types including granulocyte–monocyte progenitor cells (GMP), megakaryocyte–erythroid progenitor cells (MEP), erythroid progenitor cells (Pro‐E), HSCs, erythroid burst forming unit (BFU‐E), erythroid colony forming unit (CFU‐E), lymphocytes, basophils, mononuclear phagocytes (MPs), and megakaryocytes (Figure 4A,B; Figure S4A).

Comparative analyses revealed significant reductions in the proportions of six hematopoietic cell populations in *Dcaf13^CKO^* embryos, including HSCs, GMPs, MEPs, lymphocytes, basophils, and MPs. In contrast, the BFU‐E, CFU‐E, and Megakaryocyte compartments exhibited expansion (Figure 4C). To further dissect lineage‐specific alterations, we performed sub‐clustering and refined trajectory analysis of the lymphoid, myeloid, and erythroid branches. Pseudotime analysis revealed that *Dcaf13^CKO^* HSCs exhibited impaired differentiation toward lymphoid and myeloid lineages, but showed a skewed fate commitment toward megakaryocytes (Figure 4D–F; Figure S4B–D). Further analyses of differentially expressed genes (DEGs) identified 617 DEGs (389 upregulated and 228 downregulated) between HSCs from *Dcaf13^fl/fl^* and *Dcaf13^CKO^* embryos. Notable differences included the upregulation of cell cycle inhibitory genes (e.g., *Cdkn1a*) and pro‐apoptotic regulators (e.g., *Bax, Ccng1*), whereas *Myc* expression, a key transcription factor regulating HSC stemness, was downregulated (Figure 4G). Moreover, using single‐cell transcriptome data to more broadly profile cell‐cycle changes across HSPC subsets revealed significant reductions in S‐phase cells in the HSC and MEP subsets of *Dcaf13^CKO^* embryos (Figure 4J). Thus, overall, the scRNA‐seq results corroborate many changes detected in our immunophenotyping data, providing additional nuanced findings.

Applying the DEGs to GO enrichment analysis revealed that the upregulated genes in *Dcaf13^CKO^* HSCs were strongly associated with p53 signaling activation (Figure 4H) while the downregulated genes produced enrichments associated with DNA conformation changes and ribosome biogenesis (Figure 4I). Alternative bioinformatic interrogation using GSEA analysis also uncovered increased apoptosis and p53 pathway signatures in *Dcaf13^CKO^* HSCs and MEPs (Figure 4K,L; Figure S4E). Using the KEGG ribosome gene set as a unified metric, we further compared expression levels of all signature genes between *Dcaf13^fl/fl^* and *Dcaf13^CKO^* embryos, finding a significant downregulation of ribosome biogenesis capacity in the *Dcaf13^CKO^* HSC and MEP clusters (Figure 4M; Figure S4F). GO enrichment analysis of GMP down‐regulated genes also revealed an enrichment of myeloid differentiation and ribosome in *Dcaf13^CKO^* embryos (Figure S4G).

Together, these results suggest P53 signaling pathway activation, along with impaired ribosome biogenesis are features of *Dcaf13*‐deficient HSCs, potentially accounting for the failure of hematopoiesis.

### 2.5 P53 Knockout Partially Rescues the Cell Number and Cell Cycle Distribution of *Dcaf13^IKO^* HSCs

We next investigated whether p53 activation contributes to the hematopoietic defects observed following Dcaf13 knockout. qPCR analyses showed that the expressions of key p53 target genes including *Bax*, *P21*, *Ccng1*, *Fas* and *Puma* were significantly increased in *Dcaf13^CKO^* and *Dcaf13^IKO^* Lin^−^ cells (Figure S5A,B). Consistently, increased p53 protein levels were detected in *Dcaf13^IKO^* BM, whereas *Trp53* mRNA levels remained unchanged (Figure S5C), together substantiating p53 pathway activation in Dcaf13 knockout HSPCs. The lack of altered *Trp53* mRNA further suggested that Dcaf13 influences the post‐translational regulation of p53. Notably, a recent study showed that DCAF13 binds directly to p53 and mediates its ubiquitination, promoting its degradation in lung adenocarcinoma cells [31]. In line with this finding, we observed that DCAF13 knockdown in U2OS cells led to a marked increase in p53 protein stability (Figure S5D), consistent with a role for DCAF13 in modulating p53 turnover. Together, these data suggest that loss of Dcaf13 in HSPCs leads to stabilization and activation of p53.

**FIGURE 5:**
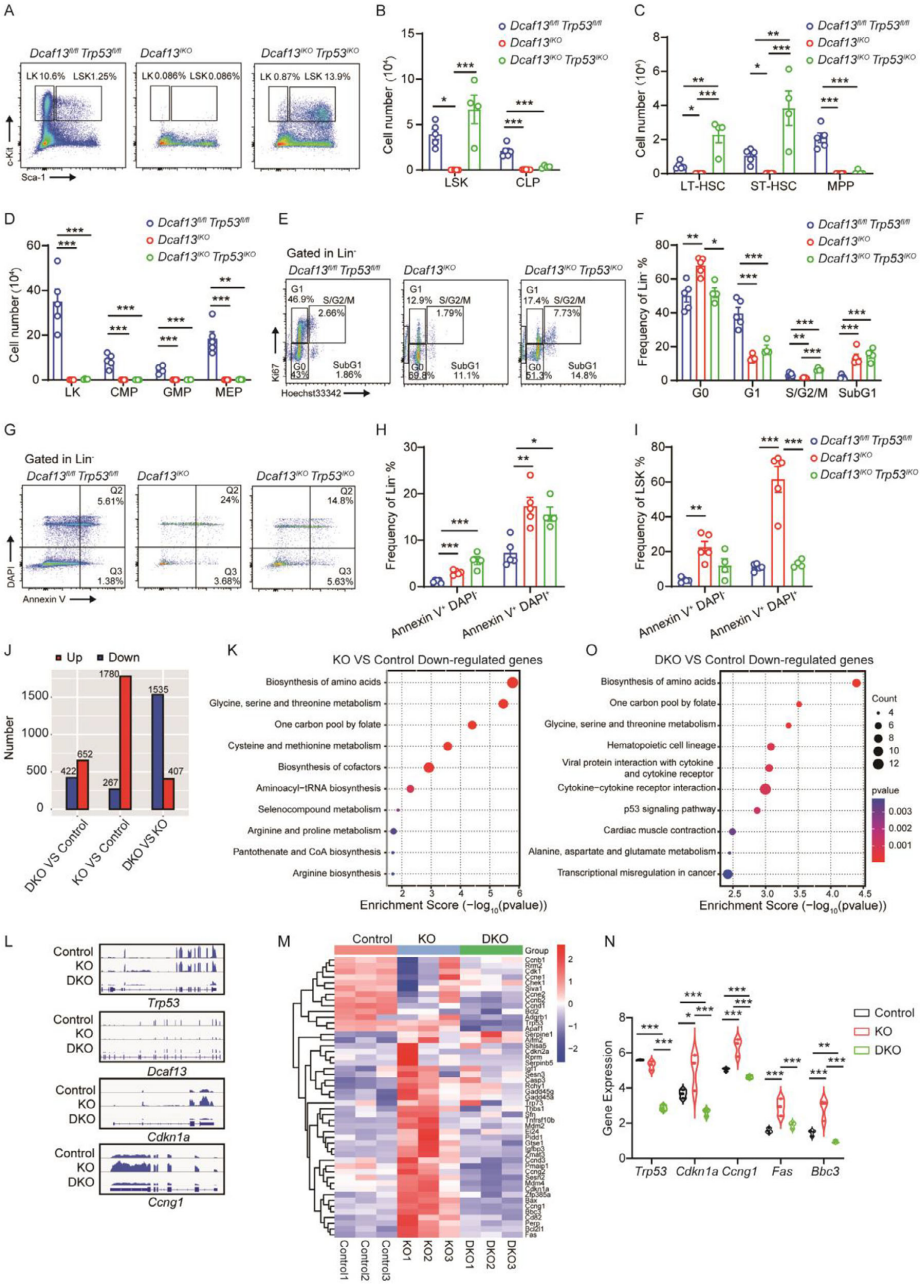
p53 double knockout partially rescues the phenotypic and transcriptomic abnormalities caused by DCAF13 depletion. (A) FACS analysis of LSK cells in the BM of *Dcaf13^fl/fl^ Trp53^fl/fl^* (Control), *Dcaf13^IKO^* (KO), and *Dcaf13^IKO^ Trp53^IKO^* (DKO) mice. (B, C) Cell number of LSK, LT‐HSC, ST‐HSC, MPP, and CLP in three group mice; n = 4–5. (D) Cell number of LK, CMP, GMP, and MEP in the BM of three group mice. (E) Ki67 staining of Lin^−^ cells in the BM of three group mice. (F) The frequencies of G0, G1, and S/G2/M phases in Lin^−^ cells are shown; n = 4–5. (G–I) Apoptosis analysis of LSKs and Lin^−^ cells using Annexin V and DAPI was performed in *Dcaf13^fl/fl^ Trp53^fl/fl^*, *Dcaf13^IKO,^* and *Dcaf13^IKO^ Trp53^IKO^* mice; n = 4‐5. (J) Numbers of differentially expressed genes (DEGs) from pairwise comparisons between Control, KO and DKO mice. |log_2_FC| > 1 and adjusted *p*‐value < 0.05. (K) Gene Ontology (GO) enrichment analysis of biological processes for genes dysregulated in the KO versus control comparisons. (L) IGV visualization of gene expression levels. Expression profiles for *Trp53*, *Dcaf13*, *Cdkn1a* (*p21*), and *Ccng1* are shown. (M) Heatmap depicting the expression of genes enriched in the p53 signaling pathway across the Control, KO, and DKO groups; the pathway gene set was derived from KEGG pathway mmu04115. FPKM values were transformed to log_2_(FPKM+1) for visualization. The complete dataset is provided in Table S5. (N) Expression of significantly dysregulated p53 target genes in the KO and DKO groups relative to the Control group. These genes were upregulated in the KO group and downregulated in the DKO group, with all meeting the criteria of |log_2_FC| > 1 and adjusted *p*‐value < 0.05. (O) GO enrichment analysis of biological processes for genes dysregulated in the DKO versus control comparisons. Data are presented as Mean ± SEM. Comparisons among the three groups were analyzed by one‐way ANOVA with Tukey's post hoc test; *p < 0.05, **p < 0.01, ***p < 0.001.

Based on the preceding results, we asked whether *Trp53* ablation could alleviate hematopoietic failure induced by *Dcaf13* loss. Accordingly, we crossed *Dcaf13^fl/fl^;Mx1‐Cre* mice with *Trp53^fl/fl^* mice to generate double knockout animals (termed *Dcaf13^IKO^ Trp53^IKO^*) (Figure S5E,F). Flow cytometry analysis of HSPC subsets following pIpC treatment showed increased numbers of LSK, LT‐HSC, and ST‐HSC cells in *Dcaf13^IKO^ Trp53^IKO^* mice compared to *Dcaf13^fl/fl^ Trp53^fl/fl^* and *Dcaf13^IKO^* littermates, although the absolute number of CLPs, MPPs, LKs, CMPs, GMPs, and MEPs remained reduced (Figure 5A–D). Further cell cycle analyses revealed increased proportions of cycling (S/G2/M phase) LSK and Lin^−^ cells from the *Dcaf13^IKO^ Trp53^IKO^* mice (Figure 5E,F, Figure S5G). Moreover, while *Trp53* deletion partially reversed the elevation in apoptotic LSK cells in *Dcaf13^IKO^* mice, apoptotic levels in Lin^−^ cells of *Dcaf13^IKO^ Trp53^IKO^* mice remained significantly higher than in *Dcaf13^fl/fl^ Trp53^fl/fl^* controls (Figure 5G–I). Despite partial improvements at the HSC level, *Dcaf13^IKO^ Trp53^IKO^* mice developed pancytopenia and died within 20 days post‐pIpC injection (Figure S5H–J), indicating persistent hematopoietic failure.

To determine why Trp53 knockout leads to a partial phenotypic rescue of hematopoiesis in Dcaf13 knockout mice, we utilized RNA‐seq to profile global transcriptomic changes. For this, we compared Lin^−^ enriched cells from *Dcaf13^fl/fl^ Trp53^fl/fl^* (Control), *Dcaf13^IKO^* (KO), and *Dcaf13^IKO^ Trp53^IKO^* (DKO) mice. We observed that 2047 genes were differentially expressed between DCAF13 knockout and control HSPCs (1780 upregulated and 267 downregulated genes, respectively), with such changes being partially reversed in DKO HSPCs (Figure 5J; FigureS5K). Functional enrichment analysis of the bulk RNA‐seq data showed good concordance with the preceding single‐cell sequencing data, with DCAF13 knockout impairing amino acid synthesis and ribosome biogenesis (Figure 5K; Figure S5L). Moreover, IGV visualization demonstrated that DCAF13 knockout did not affect p53 transcript levels but rather indicated that downstream p53 target genes were induced, including Cdkn1a (p21) and Ccng1 (Figure 5L), while these transcriptional changes were partially reversed in DKO HSPCs (Figure 5L). To further assess the broader transcriptional impact, we performed heatmap analysis of normalized FPKM expression values for p53 signaling pathway genes across the three groups. This analysis revealed that the upregulation of most p53 pathway genes observed in KO cells was attenuated in DKO cells (Figure 5M). This expression pattern indicates a rescue of the p53 transcriptional response following double knockout. Consistently, significantly elevated p53 target genes in KO cells, including Cdkn1a, Ccng1, Fas, and Bbc3, were substantially reduced in DKO cells (Figure 5N). In contrast, transcriptional defects related to amino acid metabolism and ribosome biogenesis persisted in DKO cells relative to controls (Figure 5O; Figure S5M), indicating that these alterations occur independently of p53.

Collectively, these results indicate that although Trp53 deletion partially alleviates the hematopoietic defects caused by Dcaf13 loss, it is insufficient to prevent lethality in Dcaf13‐deficient mice. Our findings suggest that Dcaf13 supports HSC maintenance through both p53‐dependent and p53‐independent mechanisms. The latter likely reflects sustained impairment of ribosome biogenesis, which may contribute to the irreversible hematopoietic failure observed in the absence of Dcaf13.

### 2.6 DCAF13 Knockout Impairs Ribosome Biogenesis and Translation in HSCs

**FIGURE 6:**
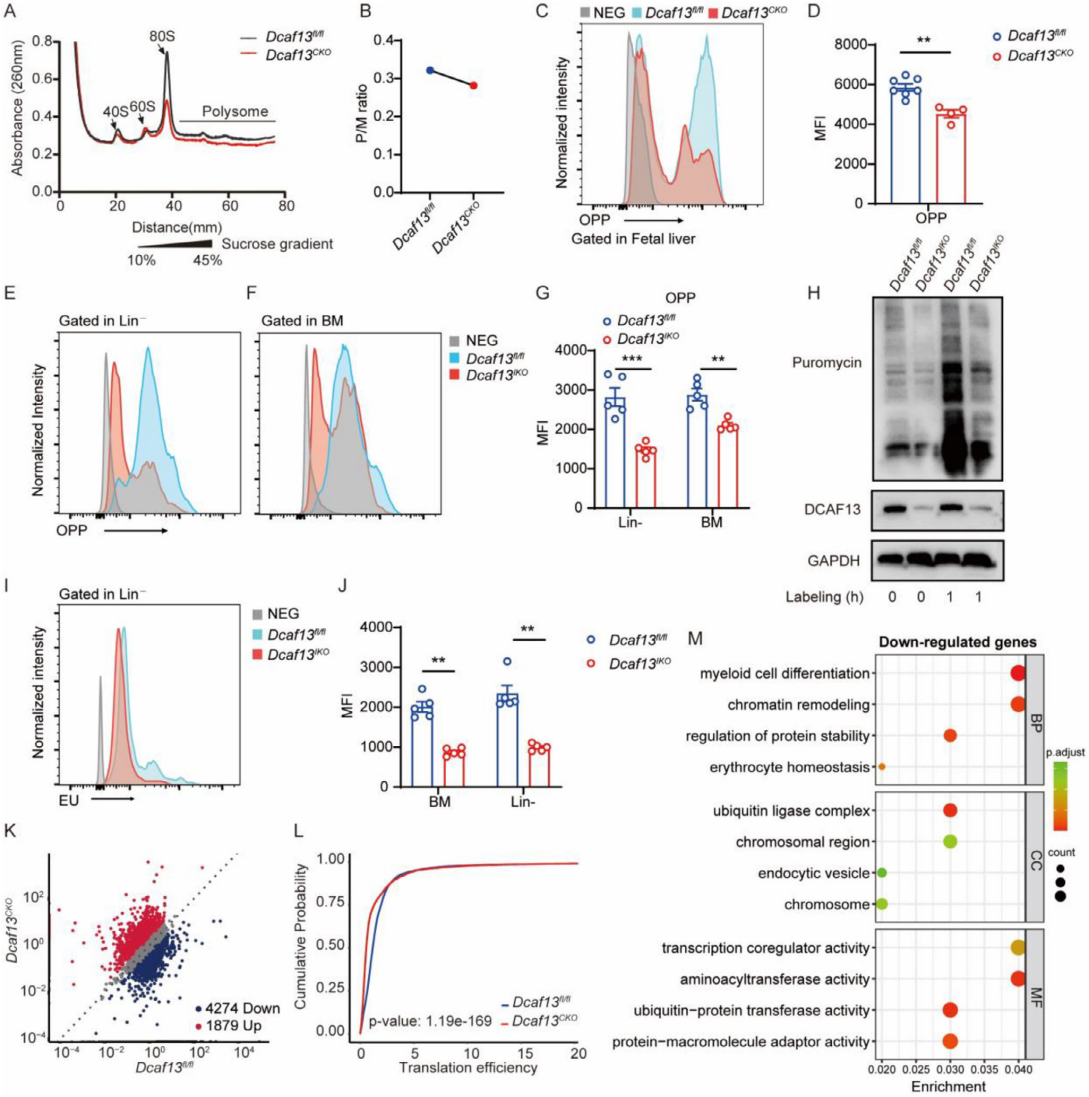
DCAF13 knockout impairs ribosome biogenesis and translation in HSCs. (A) Polysome profiling of E14.5 Lin^−^ cells from *Dcaf13^fl/fl^* and *Dcaf13^CKO^* fetal livers; Arrows indicate the large and small subunits of ribosome. (B) Quantification of polysome: monosome (P/M) ratio from polysome profiling assay. (C) Representative histograms of protein synthesis rates in fetal liver cells from *Dcaf13^fl/fl^* and *Dcaf13^CKO^* mice, as determined by Click‐iT Plus OPP protein synthesis kit. (D) Quantification of OP‐puro MFI in fetal liver cells; n = 4–7. (E–G) Quantification of protein synthesis rate in Lin^−^ and BM cells from *Dcaf13^fl/fl^* and *Dcaf13^IKO^* mice; n = 5. (H) BM cells from *Dcaf13^fl/fl^* and *Dcaf13^IKO^* mice were treated with 10µg/mL puromycin for 1 h. Puromycylation of nascent peptides was determined by Western blot. (I, J) Analysis of nascent RNA synthesis in BM and Lin^−^ cells from *Dcaf13^fl/fl^* and *Dcaf13^IKO^* mice, as determined by EU RNA Synthesis Kit with Alexa Fluor 555; n = 5. (K The scatter plot displayed the differentially translated genes identified by polysome profiling, with the polysome to monosome (P/M) ratio representing translation efficiency; |log_2_FC| ≥ 1. (L) Cumulative curve of differentially translated genes of E14.5 Lin^−^ cells from *Dcaf13^fl/fl^* and *Dcaf13^CKO^* fetal livers. (M) GO enrichment pathway of translationally downregulated genes in *Dcaf13^CKO^* Lin^−^ compared with *Dcaf13^fl/fl^* Lin^−^ cells. Data are presented as Mean ± SEM; *p < 0.05, **p < 0.01, *** *p* < 0.001, Student's t‐test.

Since Trp53 deletion only partially restored hematopoietic function in Dcaf13‐deficient mice, we next investigated whether impaired ribosome biogenesis was a potential underlying mechanism. Using polysome profiling, we monitored the levels of monosomes (40S, 60S, 80S) and polysomes in Lin^−^ cells from E14.5 fetal livers. These assays uncovered shifts involving reductions in the 80S and polysome peaks in *Dcaf13^CKO^* cells compared to controls, consequently decreasing polysome‐to‐monosome (P/M) ratios (Figure 6A,B). These alterations are consistent with reduced ribosome assembly and diminished translational capacity [32]. As confirmation, we examined global translation using OP‐Puro assays and observed that protein synthesis rates were significantly reduced in E14.5 fetal liver cells from *Dcaf13^CKO^* mice (Figure 6C,D). Moreover, similar phenotypes are evident in Lin^−^ and total BM cells from *Dcaf13^IKO^* mice (Figure 6E–G). Consistently, puromycin labeling also revealed significantly decreased translation rates in *Dcaf13^IKO^* BM cells (Figure 6H). Lastly, since pre‐rRNA synthesis and processing are the initiating and rate‐limiting steps of ribosome biogenesis [33]. We verified that global nascent RNA transcription was comparatively decreased in *Dcaf13^IKO^* Lin^−^ cells (Figure 6I,J). Thus, *Dcaf13* knockout impairs ribosome biogenesis and reduces protein translation efficiency in HSPCs, although whether specific genes or pathways were impacted required elaboration.

To address this question, we performed polysome‐seq to directly measure genome‐wide changes in translational efficiency, employing P/M ratios as our metric. As anticipated, cumulative distribution analysis revealed an overall decline in the average translation efficiency in *Dcaf13^CKO^* Lin^−^ cells compared to controls (Figure 6L). At the gene level, we identified 6153 differentially translated genes (4274 downregulated and 1879 upregulated; Figure 6K). Applying the translationally downregulated genes to enrichment analysis uncovered GO signatures for myeloid differentiation, chromatin remodeling, and erythroid homeostasis (Figure 6M) while the less number of upregulated genes were primarily related to cell migration and cell adhesion processes (Figure S6A). Thus, the failure of ribosome biogenesis in Dcaf13‐deficient cells results in a biased translational defect, disproportionately impacting mRNAs required for HSPC maintenance and function.

### 2.7 DCAF13 Regulates Efficient Ribosome Biogenesis and Translation by Stabilizing RRS1 Through K27‐Linked Non‐Degradative Ubiquitination

**FIGURE 7:**
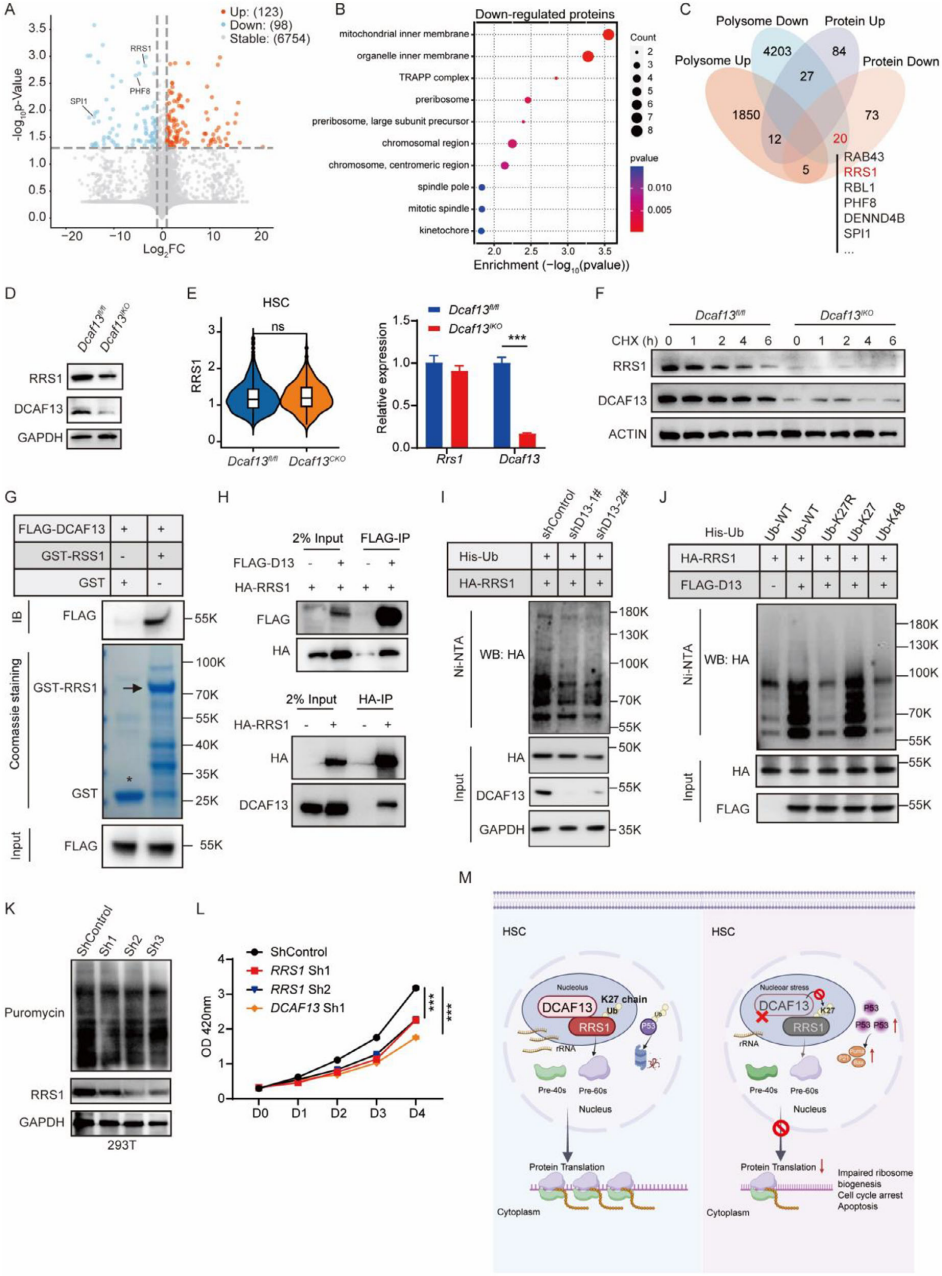
DCAF13 regulates efficient ribosome biogenesis and translation via stabilization of RRS1. (A) Volcano Plot displays differentially expressed proteins in *Dcaf13^IKO^* Lin^−^ cells compared with *Dcaf13^fl/fl^* group. (B) GO enrichment pathway of down‐regulated proteins in *Dcaf13^IKO^* Lin^−^ cells compared with *Dcaf13^fl/fl^* group. (C) Venn diagram shows the overlapped proteins between polysome profiling and proteomics. (D) Protein levels of RRS1 and DCAF13 in the BM of *Dcaf13^fl/fl^* and *Dcaf13^IKO^* mice. (E) Left panel: Violin plot shows *Rrs1* mRNA expression level in *Dcaf13^CKO^* HSC compared with *Dcaf13^fl/fl^* HSC analyzed by previous scRNA‐seq. Right panel: mRNA expression of *Rrs1* and *Dcaf13* in *Dcaf13^IKO^* Lin^−^ cells compared with *Dcaf13^fl/fl^* controls, normalized to β‐actin; n = 3. (F) RRS1 protein levels were determined in 100 µg/mL cycloheximide (CHX)‐treated *Dcaf13^fl/fl^* and *Dcaf13^IKO^* bone marrow cells for 0, 1, 2, 4, and 6 h. (G) Purified recombinant GST or GST‐RRS1 was incubated with recombinant FLAG‐DCAF13. The binding of FLAG‐DCAF13 to GST‐RRS1 was assessed by Western blot. (H) Upper panel: anti‐FLAG immunoprecipitation of lysates from 293T cells transfected with DCAF13‐3×FLAG and HA‐RRS1. Bottom panel: anti‐HA immunoprecipitation of lysates from 293T cells transfected with HA‐RRS1. (I) Ubiquitination assays in HEK293T cells after co‐transfection with HA‐RRS1 and His‐Ub after knockdown of DCAF13 using independent shRNA (shDCAF13‐1# and ‐2#). Samples recovered with Ni‐NTA and input samples were then subjected to Western blotting against anti‐HA and anti‐DCAF13 as indicated. (J) Ubiquitination of HA‐RRS1 was measured in cells co‐expressing FLAG‐DCAF13 (FLAG‐D13) with either His‐Ub WT, His‐Ub K27R, His‐Ub K27, or His‐Ub K48, respectively. (K) 293T cells were transfected with *RRS1* shRNA. 72 h after transfection, cells were treated with 10 µg/mL puromycin for 1 h. Puromycylation of nascent peptides was determined by Western blot. (L) Knockdown of *RRS1* and *DCAF13* in U2OS reduced cell growth as determined by CCK8; n = 3; (M) A model illustrating how the DCAF13‐RRS1 axis regulates HSC homeostasis. Created with BioRender.com. In normal HSCs, DCAF13 stabilizes RRS1 via K27‐linked non‐degradative ubiquitination to maintain proper ribosome biogenesis, ensuring balanced protein translation and HSC homeostasis. Once *Dcaf13* was depleted, RRS1 decreased, leading to defective ribosome assembly, activation of p53 signaling, reduced protein synthesis, and ultimately hematopoietic failure in fetal and adult mice. Data are presented as Mean ± SEM; *p < 0.05, **p < 0.01, ***p < 0.001, Student's t‐test.

It remained imperative to define the mechanism whereby DCAF13 controls ribosome homeostasis. To glean clues, proteomic analyses of Lin^−^ cells from *Dcaf13^IKO^* mice identified 123 upregulated and 98 downregulated proteins associated with *Dcaf13* deficiency (Figure 7A). Downregulated proteins were mainly associated with mitochondrial membrane, chromosomal stability along with pre‐ribosome assembly (Figure 7B), whereas the upregulated proteins aligned with cytoskeletal organization, lysosomal function, and NK cell‐mediated cytotoxicity functions (Figure S6B). To further rationalize these candidates, we intersected the proteomic and translational profiling analyses, identifying 20 downregulated and 12 upregulated proteins (Figure 7C). We found RRS1 (Regulator of Ribosome Synthesis 1), a protein directly involved in ribosome biogenesis [34, 35], was included in the list of downregulated proteins together with other potential players of interest, including the master transcriptional regulator of HSC differentiation PU.1 and the chromatin modifier PHF8 [36, 37, 38].

DCAF13 functions as a substrate receptor for the CUL4‐DDB1 E3 ubiquitin ligase complex, facilitating the ubiquitination of target proteins to control their stability and function [25, 39]. Given the connection between ribosome biogenesis and the DCAF13 knockout phenotype, we hypothesized that DCAF13 directly regulates a key factor in this pathway and prioritized RRS1 as a candidate substrate. Indeed, we found Rrs1 protein levels were markedly reduced in *Dcaf13^IKO^* BM cells compared to controls (Figure 7D), although its mRNA levels remained unchanged in *Dcaf13^CKO^* HSC and *Dcaf13^IKO^* Lin^−^ cells (Figure 7E). Additional cycloheximide (CHX) chase assays in *DCAF13^IKO^* bone marrow cells showed that DCAF13 knockout significantly reduced the half‐life of RRS1 (Figure 7F). These findings provided preliminary evidence that the post‐translational stability of RRS1 was dependent on DCAF13, but formal demonstration of direct, DCAF13‐mediated ubiquitination was required.

As the function of a substrate receptor is predicated on direct binding, we first investigated whether DCAF13 physically interacts with RRS1. Indeed, GST pull‐down assays employing purified recombinant proteins provided biochemical evidence that DCAF13 directly binds to RRS1 (Figure 7G). On this basis, we implemented cell‐based assays to investigate the DCAF13‐RRS1 regulatory relationship, a system that provides a tractable platform for experimental manipulation. Notably, the physical interaction between DCAF13 and RRS1 was readily reproduced in 293T cells after co‐transfection of epitope‐tagged DCAF13 and RRS1 constructs (Figure 7H). Importantly, the inclusion of His‐ubiquitin in these assays demonstrated that RRS1 was ubiquitinated by DCAF13 (Figure S6C) and that our data showed that knockdown of DCAF13 significantly reduced RRS1 ubiquitination (Figure 7I). Paradoxically, such ubiquitination is required to maintain RRS1 stability, revealing a non‐proteolytic mode of regulation that led us to define the specific ubiquitin chain linkages involved.

Different ubiquitin chain linkages constitute a “ubiquitin code” that leads to distinct functions. Canonical K48‐linked ubiquitination mediates proteasomal degradation, while other chain types, such as K63‐linked, K11‐linked, and K27‐linked ubiquitin, are frequently associated with non‐proteolytic functions, including the regulation of protein activity, subcellular localization, and protein–protein interactions [40, 41, 42]. We initially screened a panel of ubiquitin mutants and found that RRS1 ubiquitination by DCAF13 was specifically dependent on K27 (Figure S6D). This was subsequently verified using defined ubiquitin mutants (WT, K27, K27R, and K48), collectively demonstrating that DCAF13 specifically catalyzes K27‐linked ubiquitination of RRS1 (Figure 7J). The dramatic loss of ubiquitination upon K27 mutation confirmed that DCAF13 mediates non‐degradative K27‐linked ubiquitination of RRS1.

Finally, it was critical to relate our mechanistic observations of DCAF13‐mediated stabilization of RRS1 to DCAF13‘s role in the hematopoietic context. Notably, phenocopying a key aspect of the mouse model, puromycin labeling revealed a significant decrease in overall translation rate in RRS1‐knockdown 293T cells, whereas an increase was observed with RRS1 overexpression (Figure 7K; Figure S6E). Independent assays undertaken in U2OS cells supported these findings, with knockdown of either DCAF13 or RRS1 dampening the rates of protein synthesis with concomitant suppression of proliferation (Figure 7L; Figure S6F). Together, these findings support a model in which DCAF13 sustains translational capacity, at least in part, by stabilizing RRS1. Given the high translational demand of HSPCs, disruption of this regulatory axis likely contributes to the severe hematopoietic defects observed upon Dcaf13 deletion.

Collectively, our data demonstrate that DCAF13 maintains RRS1 stability via K27‐linked non‐degradative ubiquitination, thereby underscoring an essential role for the Dcaf13‐Rrs1 axis in maintaining HSC homeostasis by coordinating ribosome production and protein synthesis (Figure 7M). Furthermore, to validate the translational relevance of this mechanism, we found that knockdown of DCAF13 in human cord blood CD34^+^ cells (Figure S7A) significantly impaired colony‐forming capacity (Figure S7B–D), supporting a conserved and essential role for DCAF13 in mammalian HSC function.

## 3 Discussion

Our study found that conditional deletion of *Dcaf13* in both fetal and adult hematopoiesis resulted in pancytopenia arising from the profound depletion of hematopoietic progenitors. HSCs underwent cell cycle arrest, increased apoptosis, and disrupted protein synthesis to ultimately result in bone marrow failure and rapid mortality.

Mechanistically, DCAF13 knockout could activate the p53 signaling pathway and impair ribosome biogenesis. *Trp53* ablation partially restored HSC numbers and cell cycle progression but failed to prevent hematopoietic failure, indicating both p53‐dependent and p53‐independent mechanisms. We further identified that DCAF13 can interact with and stabilize RRS1 by K27‐linked non‐degradative ubiquitination, a key ribosome biogenesis factor required to sustain proper ribosome biogenesis and protein translation (Figure 7M). To directly validate whether DCAF13 acts through RRS1 to regulate HSC function, we attempted to perform a rescue experiment by overexpressing RRS1 in freshly isolated DCAF13‐deficient Lin^−^ cells. However, these knockout cells exhibited severe survival defects in vitro, with rapid cell death occurring within 24 h, making any form of genetic manipulation or extended culture technically unfeasible at this stage. Despite this limitation, we have gathered strong and multifaceted evidence supporting the functional link between DCAF13 and RRS1. We confirmed a direct physical interaction between DCAF13 and RRS1 by GST pull‑down and demonstrated that DCAF13 stabilizes RRS1 protein via K27‑linked, non‑degradative ubiquitination. Loss of DCAF13 resulted in marked downregulation of RRS1, accompanied by impaired ribosome biogenesis and global protein synthesis. Moreover, the transcriptional and functional defects observed in DCAF13‑deficient HSPCs are consistent with the established role of RRS1 in ribosome assembly. Together, our results establish a DCAF13–RRS1 regulatory axis essential for HSC homeostasis.

RRS1, in complex with RPF2, facilitates the incorporation of 5S ribonucleoprotein particles into pre‐60S ribosomal subunits [34, 35, 43]. In *Dcaf13*‐deficient cells, RRS1 protein levels were significantly reduced despite unchanged mRNA expression, and RRS1 knockdown recapitulated the translational defects seen in *Dcaf13* deletion. Our findings suggest that DCAF13 ubiquitinates and stabilizes RRS1, ensuring efficient ribosome assembly. Interestingly, proteomic analysis also revealed downregulation of key hematopoietic regulators, including PU.1 (Spi1) and PHF8, which control myeloid differentiation and chromatin stability, respectively [37, 38]. This suggests that DCAF13 may integrate ribosome biogenesis with transcriptional and epigenetic regulation, adding another layer of complexity to its role in HSC maintenance.

Recently, in hepatocellular carcinomas, RRS1 was reported to attenuate ribosomal stress through retaining RPL11 in the nucleolus, which, in turn, potentiates MDM2‐mediated ubiquitination and degradation of p53 [44]. While in our study, *Dcaf13*‐deficient cells exhibited reduced RRS1 protein level and active p53 signaling, *Trp53* ablation only partially restored HSC numbers and cell cycle progression but failed to prevent catastrophic hematopoietic failure. Recent studies found that DNA damage could induce p53‐independent apoptosis through ribosome stalling. Stalled ribosomes activated a ribotoxic stress response (RSR), where ribosomal sensing by the MAP3K. ZAKα leads to activation of p38 and JNK kinases. The activation of these kinases leads to cell cycle arrest and cell death [22, 45]. Whether *Dcaf13* depletion‐induced p53‐independent apoptosis also occurs through RSR requires further investigation.

The correlation between DCAF13 and ribosome dysregulation extends beyond hematopoietic stem cells (HSCs) and has been observed in multiple tissues. Studies have demonstrated that DCAF13 can orchestrate rRNA biogenesis via interaction with NPM1, RPA194 (subunits of RNA polymerase I), and FBL, mechanistically linking its essential role in breast cancer proliferation, T cell development, and oocyte maturation [12, 39, 46]. In our study, we also observed reduced nascent RNA synthesis in *Dcaf13^IKO^* Lin^−^ cells, suggesting that DCAF13 may partially influence ribosome biogenesis by pre‐rRNA processing and maturation in HSC through similar mechanisms.

The identification of DCAF13 as a regulator of ribosome biogenesis in HSCs has significant implications for ribosomopathies and hematopoietic disorders. Ribosomopathies are a group of congenital diseases caused by mutations in ribosomal proteins or biogenesis factors, often leading to bone marrow failure [47, 48]. Shwachman‐Diamond syndrome, for example, is associated with hematopoietic failure and increased myeloid malignancy risk [9, 11], highlighting the sensitivity of HSCs to ribosomal defects. Our findings suggest that the DCAF13‐RRS1 axis may represent a novel pathway relevant to ribosomopathies. Studies have demonstrated that ribosome biogenesis dysfunction triggers p53‐mediated apoptosis in stem cells [6], and our work further suggests that specific translational defects may underlie the cell‐type–specific vulnerability of HSCs to ribosome biogenesis disruption. Future investigations into DCAF13 and RRS1 expressions in patients with unexplained bone marrow failure syndromes may uncover new disease mechanisms.

While our data strongly support a role for DCAF13 in stabilizing RRS1 via K27‐linked polyubiquitination, the precise molecular mechanism underlying this stabilization remains incompletely defined. Given the highly dynamic nature of the ubiquitin–proteasome system, substrate fate is often dictated by competition between stabilizing and degradative ubiquitin signals, as well as by the actions of opposing E3 ligases and deubiquitinating enzymes. Future studies will be required to map the specific RRS1 lysine residue(s) targeted by DCAF13 and to determine whether K27‐linked ubiquitination sterically or functionally interferes with recruitment of E3 ligases, ubiquitin receptors, or proteasomal adaptors involved in RRS1 degradation. Future studies are essential for a complete mechanistic understanding of RRS1 regulation in hematopoiesis.

In summary, our study identifies DCAF13 as an essential regulator of HSC maintenance through the DCAF13‐RRS1 axis, which is critical for proper ribosome biogenesis and protein translation. These findings provide novel insights into the molecular mechanisms underlying HSC homeostasis and hematopoietic failure. Given the significant implications for ribosomopathies and bone marrow failure syndromes, examining the expression and function of DCAF13 and RRS1 in patients with unexplained bone marrow failure or myelodysplastic syndromes may reveal new pathogenic mechanisms and potential therapeutic targets.

## 4 Experimental Section/Method

### 4.1 Animals

*Dcaf13^fl/fl^* mice were generated using CRISPR‐Cas9, with loxP sites flanking exon 2 of the *Dcaf13* gene. All animal experiments were approved by the Animal Care and Use Committee of Zhengzhou University (ZZU‐LAC20220729) and conducted in compliance with institutional guidelines. To delete *Dcaf13* in the hematopoietic system, *Dcaf13^fl/fl^* mice were crossed with *Vav‐iCre* and *Mx1‐Cre* transgenic mice. For *Dcaf13^fl/fl^;Vav‐iCre* embryos (*Dcaf13^CKO^*), littermates including *Dcaf13^fl/+^*, *Dcaf13^fl/fl^*, and *Dcaf13^fl/+^;Vav‐iCre* were used as controls. For inducible deletion in adult mice, *Dcaf13^fl/fl^* mice were crossed with *Mx1‐Cre* transgenic mice to generate *Dcaf13^fl/fl^;Mx1‐Cre* mice. To examine potential interactions with p53, *Dcaf13^fl/fl^;Mx1‐Cre* mice were further crossed with *Trp53^fl/fl^* mice. Genotyping was performed by PCR using the primers listed in Table S1. All transgenic and conditional knockout mice were on C57BL/6‐Ly5.2 (Ly5.2, CD45.2) genetic background. Male and female mice with a comparable number and age (6–8 weeks) were randomly selected for experiments. B6.SJL‐Ly5.1 (Ly5.1, CD45.1) mice (6‐8 weeks) were used for hematopoietic transplantation assays.

### 4.2 Isolation of Hematopoietic Cells

Embryos were harvested at E12.5 and E14.5. Fetal livers were dissociated and resuspended in PBS + 2% FBS. BM cells were flushed from femurs, tibias, and fibulas, and thymus and spleen single‐cell suspensions were prepared by gentle mechanical disruption. For human HSPC isolation, umbilical cord blood samples were collected with informed consent from participants, following approval by the Institutional Ethics Committee of Henan Provincial People's Hospital (Approval No. 2024191). CD34^+^ cells were enriched using a CD34 MicroBead Kit (Miltenyi, Bergisch Gladbach, Germany).

### 4.3 Induction of Mx1‐Cre Expression

For inducible *Dcaf13* deletion in adult mice, 6‐ to 8‐week‐old *Dcaf13^fl/fl^;Mx1‐Cre* mice (hereafter referred to as *Dcaf13^IKO^*) and controls were injected intraperitoneally with poly(I:C) (pIpC, InvivoGen, Cat#tlrl‐pic) at a dose of 15 mg/kg every other day for three injections. Mice were analyzed 7 days after the first injection, unless otherwise specified.

### 4.4 Embryo Collection

Timed matings between *Dcaf13^fl/fl^* and *Dcaf13^fl/+^;Vav‐iCre* mice were set up, with the detection of a vaginal plug considered embryonic day 0.5 (E0.5). Embryos were harvested at E12.5, E14.5, E16.5, and E18.5 for analysis.

### 4.5 Complete Blood Count Analysis

Peripheral blood was collected from the retro‐orbital sinus into EDTA‐coated tubes, and complete blood counts were measured using an automated hematology analyzer (HEMAVET 950, Drew Scientific) following the manufacturer's instructions.

### 4.6 Flow Cytometry Analysis and Cell Sorting

Single cells were prepared from fetal liver, bone marrow, spleen, thymus, and peripheral blood. Analysis of HSPCs, subpopulations of mature cells, was performed as we described previously [49]. All antibodies are listed in Table S1. Donor chimerism in transplantation assays was assessed using CD45.1, CD45.2, B220, CD3, Gr‐1, and CD11b markers. For the Ki67 staining assay, cells were stained with a cell surface marker first, fixed with BD FACS lysing solution, permeabilized with BD IntraSure Kit, intracellularly stained with Ki67 antibody, and resuspended in PBS buffer. Hoechst 33342 was added prior to FACS analysis. For apoptosis staining, cells were stained with HSC markers first, then resuspended in BD Binding Buffer and stained with anti‐Annexin V (BD) for 15 min at room temperature. Cells were sorted and analyzed on an Aria flow cytometer instrument (BD Biosciences). Flow cytometry data were analyzed using FlowJo software (v10.8.1). For HSC sorting, lineage‐negative cells were first enriched using the EasySep Mouse Hematopoietic Progenitor Cell Isolation Kit [STEMCELL, Cat#19856A]. The cells were then stained with surface markers for 30 min at 4°C and washed with PBS + 2%FBS buffer. Subsequently, samples were incubated with fluorescent dye‐conjugated antibodies for 30 min and analyzed by flow cytometry.

### 4.7 Colony Formation Assays

Fetal liver or BM cells (2 × 10^4^) were plated in methylcellulose medium (MethoCult GF M3434, StemCell Technologies) and cultured at 37°C, 5% CO_2_. Colonies were counted after 12–14 days.

### 4.8 Transplantation Assays

For competitive transplantation, 3 × 10^5^ fetal liver cells (CD45.2) from E14.5 *Dcaf13^fl/fl^* or *Dcaf13^CKO^* embryos were mixed with 5 × 10^5^ bone marrow cells (CD45.1) and intravenously injected into lethally irradiated CD45.1 recipient mice. 5 × 10^5^ BM cells (CD45.2) from *Dcaf13^fl/fl^* or *Dcaf13^fl/fl^;Mx1‐Cre* mice (pIpC‐untreated) were mixed with 5 × 10^5^ BM cells (CD45.1) and injected into lethally irradiated CD45.1 recipients. BM chimerism was monitored by fluorescence‐activated cell sorting of peripheral blood (PB) at 4 weeks, and then the mice were injected 3 times with pIpC (InvivoGen). For secondary transplantation, 2 × 10^6^ BM cells from primary recipients were injected into lethally irradiated CD45.1 recipient mice. For LSK competitive transplantation, 1000 sorted LSK cells (CD45.2) from untreated *Dcaf13^fl/fl^* or *Dcaf13^fl/fl^; Mx1‐Cre* mice were mixed with an equal number of competitor bone marrow cells (CD45.1) and transplanted into lethally irradiated CD45.1 recipients. The recipients were injected with pIpC four weeks post‐transplantation.

### 4.9 Histological Analysis

Fetal livers, spleens, thymuses, femurs, and sternums were fixed in 4% paraformaldehyde overnight at 4°C. Bones were decalcified in 10% EDTA for 7 days, dehydrated, embedded in paraffin, sectioned (4 µm), and stained with H&E.

### 4.10 Immunohistochemistry

Paraffin sections were deparaffinized, rehydrated, and subjected to antigen retrieval in Citrate Antigen Retrieval Solution. Endogenous peroxidase was blocked with 3% hydrogen peroxide, and sections were incubated with primary antibodies overnight at 4°C, followed by high‐ionic‐strength reaction buffer treatment and HRP‐conjugated secondary antibodies. Signals were developed with DAB substrate [Zsbio], counterstained with hematoxylin, and mounted with neutral balsam.

### 4.11 RNA Isolation and Quantitative RT‐PCR

Total RNA was extracted using TRIzol reagent [Thermo fisher], reverse‐transcribed with the SweScript All‐in‐One RT SuperMix [Servicebio], and analyzed by quantitative PCR with SYBR Green PCR Master Mix [Servicebio] on a StepOne Plus real‐time PCR detector (applied biosystems). Relative gene expression was calculated using the 2^−ΔΔCt^ method with β‐actin as an internal control. All primers used in this study are listed in Table S1.

### 4.12 Single Cell RNA‐Sequencing and Analysis

Single‐cell suspensions were prepared by collecting E14.5 fetal liver cells from 2 pairs of *Dcaf13^fl/fl^* and *Dcaf13^CKO^* embryos, then enriched with Easysep mouse hematopoietic progenitor cell isolation kit. The SCOPE‐chip, which utilizes a microfluidics system, was used for comprehensive single‐cell sequencing analysis. The cells were labeled with barcodes with a unique molecular identifier (UMI) sequence. The reverse transcription and template switch steps were then performed. Finally, the cDNA libraries were generated and sequenced on the Illumina platform. The raw sequencing reads were processed using CeleScope of Singleron. We sequenced and retained 65478 cells after the quality‐control process, including 34756 cells for *Dcaf13^fl/fl^* embryos and 30722 for *Dcaf13^CKO^* embryos. PCA (Principal Component Analysis) and Louvain analysis were performed to group cells based on the top 50 PCs. Cell types were annotated by Cell ID. t‐SNE and UMAP were used to visualize. We identified 14 groups and performed differential expression analysis and functional enrichment analysis for each group. Cell type annotation and differentially expressed genes (DEGs) were listed in Table S2. For bulk RNA‐seq, we enriched Lin^−^ cells from *Dcaf13^fl/fl^ Trp53^fl/fl^*, *Dcaf13^IKO,^* and *Dcaf13^IKO^ Trp53^IKO^* mice. Total RNA was extracted using TRIzol according to the manufacturer's instructions and processed in a standard mRNA library construction pipeline. We applied a cutoff of |log_2_FC| > 1 and adjusted *p*‐value ≤ 0.05 to identify differentially expressed genes. The data have been submitted to the China National Genomics Data Center.

### 4.13 Polysome Profiling

Lin^−^ cells were enriched from fetal livers of E14.5 *Dcaf13^fl/fl^* and *Dcaf13^CKO^* embryos. 2 × 10^6^ cells were collected and treated with cycloheximide at 100 µg/mL for 15 min at 37°C. Cells were then washed twice in ice‐cold PBS. Cell pellets were lysed in polysome lysis buffer. The lysate was cleared by a series of centrifugations, loaded on a 10%–45% sucrose gradient, and ultra‐centrifuged at 40 000 rpm for 3 h at 4°C with a Beckman Optima XE‐100 centrifuge. Then the gradient was fractionated using Gradient Profiler (BioComp) and OD_254_ was measured. RNA of the polysome and non‐polysome fractions was extracted using a standard phenol‐chloroform method. Polysome‐seq was performed by Qingze Biotech (China). Threshold for differentially translated gene**s**: |log_2_FC| ≥ 1. Differentially translated genes were listed in Table S3.

### 4.14 OP‐Puro (O‐Propargyl‐Puromycin) Incorporation Assay

Total BM cells were harvested and live‐stained with cell surface markers for HSCs. Cells were then incubated with OP‐Puro (20 µm, 1 h), fixed, permeabilized, and analyzed by flow cytometry following the manufacturer's protocol of the Click‐iT Plus OPP Alexa Flour 488 Protein Synthesis Assay Kit [Thermo Fisher]. The translation rate was also measured using the SUnSET assay, as previously reported [50, 51]. 1 million cells were seeded onto a 6‐well plate and treated with puromycin (Beyotime) at 10 µg mL^−1^ for 30 min or 1 h at 37°C. Cells were then collected, and puromycin‐incorporated proteins were detected by Western blot using anti‐puromycin antibody (Millipore).

### 4.15 Nascent RNA Incorporation Assay

BM cells were first stained with cell surface markers, then incubated for 1 h with 1 mm ethynyl uridine (EU) at 37°C, fixed, and permeabilized. Nascently synthesized RNA was detected using the Click‐iT EU‐555 RNA Synthesis Kit (Beyotime) according to the protocol and analyzed by flow cytometry.

### 4.16 Co‐Immunoprecipitation

For the Co‐IP assay, 293T cells were transfected with the tagged plasmid. After 48 h of transfection, cells were lysed in IP lysis buffer containing 1% NP40, 10% Glycerol, 135 mm NaCl, 20 mm Tris‐HCl, pH 8.0, supplemented with protease and phosphatase inhibitors as previously described [49]. Whole cell lysates were pre‐cleared and incubated with FLAG‐beads or HA‐beads overnight (O/N) at 4°C, then washed three times. Subsequently, beads were boiled directly in 2x SDS loading buffer and analyzed by SDS‐PAGE.

### 4.17 GST Pull‐Down Assay

GST‐tagged RRS1 was expressed in Escherichia coli BL21 by induction with 100 mm Isopropyl β‐D‐1‐thiogalactopyranoside for 16 h at 18°C and affinity‐purified using Anti‐GST Magnetic Beads (Beyotime). GST‐fusion proteins were incubated with FLAG‐DCAF13 in pull‐down buffer for 4 h at 4°C. After incubation, the input and bead‐bound proteins were analyzed using an immunoblotting assay.

### 4.18 Ubiquitination Assays

HEK293T cells were cotransfected with His‐ubiquitin, HA‐RRS1, and FLAG‐DCAF13. After 48 h, the cells were lysed in 8 m urea lysis buffer as previously described [52]. The lysates were sonicated and incubated with Ni‐NTA beads for 3 h at 25°C to capture ubiquitinated proteins. After washing, the proteins were eluted and analyzed by immunoblotting using the indicated antibodies.

### 4.19 Cycloheximide (CHX) Chase Assay

CHX chase assay was used to determine the half‐life of RRS1. 6‐ to 8‐week‐old *Dcaf13^fl/fl^;Mx1‐Cre* mice and controls were injected intraperitoneally with pIpC at a dose of 15 mg/kg every other day for three injections. On day 7 after the first injection, mice were sacrificed. BM cells were individually seeded in 6‐well plates and treated with cycloheximide (CHX; 100 µg/mL) for 0, 1, 2, 4, and 6 h. Next, Western blot was employed to analyze the expression of RRS1 protein.

### 4.20 Quantitative Proteomics

Lin^−^ cells were enriched from *Dcaf13^fl/fl^* and *Dcaf13^IKO^* mice; 2 × 10^6^ cells were collected and washed twice in ice‐cold PBS. Proteins were extracted and enzymatically digested from cell pellets, followed by optical desalting and high‐resolution mass spectrometry analysis. Liquid chromatography‐mass spectrometry (LC‐MS) was used to quantify the enzymatically digested peptides. MS/MS spectra were searched using MASCOT engine (Matrix Science, version 2.2) against Universal Protein (UniProt) database. Threshold for differentially expressed protein**s**: |log_2_FC| **>** 1 and *p*‐value ≤ 0.05. Details of differentially expressed proteins identified with mass spectrometry are listed in Table S4.

### 4.21 Statistical Analysis

Data was analyzed using Prism 10.0 (GraphPad Software). Survival analysis was performed using the Kaplan‐Meier test in Prism 10.0. Data represent at least three independent biological replicates. Distribution was tested by Shapiro‐Wilk normality test. When parameters followed a Gaussian distribution, Student's t test was used to compare two groups; for more than two groups, the one‐way ANOVA was used; otherwise, the Mann–Whitney test was employed. Significance was set at *p* < 0.05 (asterisks indicate *p < 0.05, **p < 0.01, and ***p < 0.001). Sample size ‘n’ indicates biological replicates.

## Author Contributions

S. Chen, D. Wei, and Z. Zhu conceived and supervised the project and revised the paper. M. Li and Y. Wu designed and performed the experiments, analyzed the data, and wrote the manuscript. S. Zhou, J. Wei, Z. Wang, P. Tang, L. Liu, Q. Zhang, D. Shi, R. Zhang, Y. Wang, and H. Zhao helped with the in vivo experiments and flow cytometry analysis. Q. Zhang, S. Yuan, S. Wang, Y. Zhang, and G. Liu contribute to the related discussion. X. Zhang, X. Chen, J. Li, R. Throne, and X. Shi contributed to the reagents, data analysis, and paper revision.

## Funding

This work was supported by the National Natural Science Foundation of China (Nos. 82300135 to L.M., U2004138, 32570881 to S.C., 82470184 to Z. Z., 81872393 to D.W., 82400143 to S.W.), the Henan Province and Ministry of Health of Medical Science and Technology Program (SBGJ202403001 to L.M.), and the Science and Technology Research Project of Henan Province (242102311148 to L.M., 242102310029 to Q.Z.).

## Conflicts of Interest

The authors declare no conflicts of interest.

## Supporting information




**Supporting File 1**: advs74611‐sup‐0001‐SuppMat.docx.

**Supporting File 2**: advs74611‐sup‐0002‐TableS1‐S5.xlsx.

## Data Availability

The data that support the findings of this study are available from the corresponding author upon reasonable request.

## References

[1] S. H. Orkin and L. I. Zon , “Hematopoiesis: An Evolving Paradigm for Stem Cell Biology,” Cell 132, no. 4 (2008): 631–644, 10.1016/j.cell.2008.01.025. 18295580 PMC2628169

[2] A. Mendelson and P. S. Frenette , “Hematopoietic Stem Cell Niche Maintenance During Homeostasis and Regeneration,” Nature Medicine 20, no. 8 (2014): 833–846, 10.1038/nm.3647. PMC4459580 25100529

[3] S. Doulatov , F. Notta , E. Laurenti , and J. E. Dick , “Hematopoiesis: A Human Perspective,” Cell stem cell 10, no. 2 (2012): 120–136, 10.1016/j.stem.2012.01.006. 22305562

[4] R. A. Signer , J. A. Magee , A. Salic , and S. J. Morrison , “Haematopoietic Stem Cells Require a Highly Regulated Protein Synthesis Rate,” Nature 509, no. 7498 (2014): 49–54, 10.1038/nature13035. 24670665 PMC4015626

[5] R. K. Khajuria , M. Munschauer , J. C. Ulirsch , et al., “Ribosome Levels Selectively Regulate Translation and Lineage Commitment in Human Hematopoiesis,” Cell 173, no. 1 (2018): 90–103.e119, 10.1016/j.cell.2018.02.036. 29551269 PMC5866246

[6] H. Wang , Z. Zhang , C. Han , et al., “SNORD113–114 Cluster Maintains Haematopoietic Stem Cell Self‐Renewal via Orchestrating the Translation Machinery,” Nature Cell Biology 27, no. 2 (2025): 246–261, 10.1038/s41556-024-01593-7. 39890952

[7] Z. Zheng , S. Yang , F. Gou , et al., “The ATF4‐RPS19BP1 Axis Modulates Ribosome Biogenesis to Promote Erythropoiesis,” Blood 144, no. 7 (2024): 742–756, 10.1182/blood.2023021901. 38657191

[8] A. J. Warren , “Molecular Basis of the Human Ribosomopathy Shwachman‐Diamond Syndrome,” Advances in Biological Regulation 67 (2018): 109–127, 10.1016/j.jbior.2017.09.002. 28942353 PMC6710477

[9] C. R. Reilly and A. Shimamura , “Predisposition to Myeloid Malignancies in Shwachman‐Diamond Syndrome: Biological Insights and Clinical Advances,” Blood 141, no. 13 (2023): 1513–1523, 10.1182/blood.2022017739. 36542827 PMC10082379

[10] L. D. Costa , T. Leblanc , and N. Mohandas , “Diamond‐Blackfan Anemia, Blood,” The Journal of the American Society of Hematology 136, no. 11 (2020): 1262–1273, 10.1182/blood.2019000947. PMC7483438 32702755

[11] N. Kawashima , U. Oyarbide , M. Cipolli , V. Bezzerri , and S. J. Corey , “Shwachman‐Diamond Syndromes: Clinical, Genetic, and Biochemical Insights From the Rare Variants,” Haematologica 108, no. 10 (2023): 2594–2605, 10.3324/haematol.2023.282949. 37226705 PMC10543188

[12] J. Zhang , Y. L. Zhang , L. W. Zhao , et al., “Mammalian Nucleolar Protein DCAF13 Is Essential for Ovarian Follicle Maintenance and Oocyte Growth by Mediating rRNA Processing,” Cell Death & Differentiation 26, no. 7 (2019): 1251–1266, 10.1038/s41418-018-0203-7. 30283081 PMC6748096

[13] J. Lee and P. Zhou , “DCAFs, the Missing Link of the CUL4‐DDB1 Ubiquitin Ligase,” Molecular Cell 26, no. 6 (2007): 775–780, 10.1016/j.molcel.2007.06.001. 17588513

[14] S. Jackson and Y. Xiong , “CRL4s: The CUL4‐RING E3 Ubiquitin Ligases,” Trends in Biochemical Sciences 34, no. 11 (2009): 562–570, 10.1016/j.tibs.2009.07.002. 19818632 PMC2783741

[15] S. Angers , T. Li , X. Yi , M. J. MacCoss , R. T. Moon , and N. Zheng , “Molecular Architecture and Assembly of the DDB1–CUL4A Ubiquitin Ligase Machinery,” Nature 443, no. 7111 (2006): 590–593, 10.1038/nature05175. 16964240

[16] S. Boulon , B. J. Westman , S. Hutten , F. M. Boisvert , and A. I. Lamond , “The Nucleolus Under Stress,” Molecular Cell 40, no. 2 (2010): 216–227, 10.1016/j.molcel.2010.09.024. 20965417 PMC2987465

[17] M. C. Lafita‐Navarro and M. Conacci‐Sorrell , “Nucleolar Stress: From Development to Cancer,” Seminars in Cell & Developmental Biology 136 (2023): 64–74, 10.1016/j.semcdb.2022.04.001. 35410715 PMC9883801

[18] M. Dumble , L. Moore , S. M. Chambers , et al., “The Impact of Altered p53 Dosage on Hematopoietic Stem Cell Dynamics During Aging,” Blood 109, no. 4 (2007): 1736–1742, 10.1182/blood-2006-03-010413. 17032926 PMC1794064

[19] Y. Liu , S. E. Elf , Y. Miyata , et al., “p53 Regulates Hematopoietic Stem Cell Quiescence,” Cell Stem Cell 4, no. 1 (2009): 37–48, 10.1016/j.stem.2008.11.006. 19128791 PMC2839936

[20] A. James , Y. Wang , H. Raje , R. Rosby , and P. DiMario , “Nucleolar Stress With and Without p53,” Nucleus 5, no. 5 (2014): 402–426, 10.4161/nucl.32235. 25482194 PMC4164484

[21] T. Teng , G. Thomas , and C. A. Mercer , “Growth Control and Ribosomopathies,” Current Opinion in Genetics & Development 23, no. 1 (2013): 63–71, 10.1016/j.gde.2013.02.001. 23490481

[22] N. J. Boon , R. A. Oliveira , P. R. Körner , et al., “DNA Damage Induces p53‐Independent Apoptosis Through Ribosome Stalling,” Science 384, no. 6697 (2024): 785–792, 10.1126/science.adh7950. 38753784

[23] A. Stedman , S. Beck‐Cormier , M. L. Bouteiller , et al., “Ribosome Biogenesis Dysfunction Leads to p53‐Mediated Apoptosis and Goblet Cell Differentiation of Mouse Intestinal Stem/Progenitor Cells,” Cell Death & Differentiation 22, no. 11 (2015): 1865–1876, 10.1038/cdd.2015.57. 26068591 PMC4648334

[24] A. Ogawa , K. Izumikawa , S. Tate , et al., “SLFN11‐Mediated Ribosome Biogenesis Impairment Induces TP53‐Independent Apoptosis,” Molecular Cell 85, no. 5 (2025): 894–912.e810, 10.1016/j.molcel.2025.01.008. 39909041 PMC11890970

[25] Y. L. Zhang , L. W. Zhao , J. Zhang , et al., “DCAF13 promotes Pluripotency by Negatively Regulating SUV39H1 Stability During Early Embryonic Development,” The EMBO Journal 37, no. 18 (2018): EMBJ201898981, 10.15252/embj.201898981. PMC6138440 30111536

[26] Q. Zhou , X. Li , N. Wang , et al., “DCAF13 is Essential for Mouse Uterine Function and Fertility,” Cell Death Discovery 11, no. 1 (2025): 359, 10.1038/s41420-025-02583-w. 40750792 PMC12316921

[27] X. Gao , F. Hong , Z. Hu , et al., “ABC Portal: A Single‐Cell Database and Web Server for Blood Cells,” Nucleic Acids Research 51, no. D1 (2023): D792–D804, 10.1093/nar/gkac646. 35920330 PMC9825444

[28] T. Yokomizo , T. Ideue , S. Morino‐Koga , et al., “Independent Origins of Fetal Liver Haematopoietic Stem and Progenitor Cells,” Nature 609, no. 7928 (2022): 779–784, 10.1038/s41586-022-05203-0. 36104564

[29] M. H. Baron , J. Isern , and S. T. Fraser , “The Embryonic Origins of Erythropoiesis in Mammals,” Blood 119, no. 21 (2012): 4828–4837, 10.1182/blood-2012-01-153486. 22337720 PMC3367890

[30] W. Wei , X. Gao , J. Qian , et al., “Beclin 1 Prevents ISG15‐Mediated Cytokine Storms to Secure Fetal Hematopoiesis and Survival,” Journal of Clinical Investigation 135, no. 3 (2025), 10.1172/jci177375. PMC11785930 39589832

[31] S. Wei , J. Xing , J. Chen , et al., “DCAF13 Inhibits the p53 Signaling Pathway by Promoting p53 Ubiquitination Modification in Lung Adenocarcinoma,” Journal of Experimental & Clinical Cancer Research 43, no. 1 (2024): 3, 10.1186/s13046-023-02936-2. 38163876 PMC10759521

[32] A. P. Schuller and R. Green , “Roadblocks and Resolutions in Eukaryotic Translation,” Nature Reviews Molecular Cell Biology 19, no. 8 (2018): 526–541, 10.1038/s41580-018-0011-4. 29760421 PMC6054806

[33] K. Dörner , C. Ruggeri , I. Zemp , and U. Kutay , “Ribosome Biogenesis Factors‐From Names to Functions,” The EMBO Journal 42, no. 7 (2023): 112699, 10.15252/embj.2022112699. PMC10068337 36762427

[34] S. Kharde , F. R. Calviño , A. Gumiero , K. Wild , and I. Sinning , “The Structure of Rpf2–Rrs1 Explains Its Role in Ribosome Biogenesis,” Nucleic Acids Research 43, no. 14 (2015): 7083–7095, 10.1093/nar/gkv640. 26117542 PMC4538828

[35] N. Asano , K. Kato , A. Nakamura , K. Komoda , I. Tanaka , and M. Yao , “Structural and Functional Analysis of the Rpf2‐Rrs1 Complex in Ribosome Biogenesis,” Nucleic Acids Research 43, no. 9 (2015): 4746–4757, 10.1093/nar/gkv305. 25855814 PMC4482071

[36] P. Burda , P. Laslo , and T. Stopka , “The Role of PU.1 and GATA‐1 Transcription Factors During Normal and Leukemogenic Hematopoiesis,” Leukemia 24, no. 7 (2010): 1249–1257, 10.1038/leu.2010.104. 20520638

[37] H. Iwasaki , C. Somoza , H. Shigematsu , et al., “Distinctive and Indispensable Roles of PU.1 in Maintenance of Hematopoietic Stem Cells and Their Differentiation,” Blood 106, no. 5 (2005): 1590–1600, 10.1182/blood-2005-03-0860. 15914556 PMC1895212

[38] J. E. Kim , X. Pan , K. Y. Tse , H. H. Chan , C. Dong , and M. S. Y. Huen , “PHF8 Facilitates Transcription Recovery Following DNA Double‐Strand Break Repair,” Nucleic Acids Research 52, no. 17 (2024): 10297–10310, 10.1093/nar/gkae661. 39087553 PMC11417394

[39] Z. Z. Yang , B. Yang , H. Yan , et al., “DCAF13‐Mediated K63‐Linked Ubiquitination of RNA Polymerase I Promotes Uncontrolled Proliferation in Breast Cancer,” Nature Communications 16, no. 1 (2025): 557, 10.1038/s41467-025-55851-9. PMC11718263 39788980

[40] S. Chen , J. Lin , Z. Yang , et al., “TRIM24‐Mediated K27‐Linked Ubiquitination of ULK1 Alleviates Energy Stress‐Induced Autophagy and Promote Prostate Cancer Growth in the Context of SPOP Mutation,” Cell Death and Differentiation (2025): 1–6, 10.1038/s41418-025-01582-9. PMC13035862 40975747

[41] H. Rho , S. Kim , S. U. Kim , et al., “CHIP Ameliorates Nonalcoholic Fatty Liver Disease via Promoting K63‐ and K27‐linked STX17 Ubiquitination to Facilitate Autophagosome‐Lysosome Fusion,” Nature Communications 15, no. 1 (2024): 8519, 10.1038/s41467-024-53002-0. PMC11445385 39353976

[42] D. Zhao , G. Zhong , J. Li , et al., “Targeting E3 Ubiquitin Ligase WWP1 Prevents Cardiac Hypertrophy Through Destabilizing DVL2 via Inhibition of K27‐Linked Ubiquitination,” Circulation 144, no. 9 (2021): 694–711, 10.1161/circulationaha.121.054827. 34139860

[43] J. Zhang , P. Harnpicharnchai , J. Jakovljevic , et al., “Assembly Factors Rpf2 and Rrs1 Recruit 5S rRNA and Ribosomal Proteins rpL5 and rpL11 Into Nascent Ribosomes,” Genes & Development 21, no. 20 (2007): 2580–2592, 10.1101/gad.1569307. 17938242 PMC2000323

[44] P. Cao , A. Yang , P. Li , et al., “Genomic Gain of RRS1 Promotes Hepatocellular Carcinoma Through Reducing the RPL11‐MDM2‐p53 Signaling,” Science Advances 7, no. 35 (2021): abf4304, 10.1126/sciadv.abf4304. PMC8386927 34433556

[45] A. C. Vind , A. V. Genzor , and S. Bekker‐Jensen , “Ribosomal Stress‐Surveillance: Three Pathways Is a Magic Number,” Nucleic Acids Research 48, no. 19 (2020): 10648–10661, 10.1093/nar/gkaa757. 32941609 PMC7641731

[46] L. Zhou , S. Wang , W. Hu , et al., “T Cell Proliferation Requires Ribosomal Maturation in Nucleolar Condensates Dependent on DCAF13,” Journal of Cell Biology 222, no. 10 (2023): 202201096, 10.1083/jcb.202201096. PMC10450623 37615668

[47] E. W. Mills and R. Green , “Ribosomopathies: There's Strength in Numbers,” Science 358, no. 6363 (2017): aan2755, 10.1126/science.aan2755. 29097519

[48] A. Narla and B. L. Ebert , “Ribosomopathies: Human Disorders of Ribosome Dysfunction,” Blood 115, no. 16 (2010): 3196–3205, 10.1182/blood-2009-10-178129. 20194897 PMC2858486

[49] M. Li , C. Qiu , Y. Bian , et al., “SETD5 Modulates Homeostasis of Hematopoietic Stem Cells by Mediating RNA Polymerase II Pausing in Cooperation With HCF‐1,” Leukemia 36, no. 4 (2022): 1111–1122, 10.1038/s41375-021-01481-1. 34853439 PMC8979820

[50] E. K. Schmidt , G. Clavarino , M. Ceppi , and P. Pierre , “SUnSET, a Nonradioactive Method to Monitor Protein Synthesis,” Nature Methods 6, no. 4 (2009): 275–277, 10.1038/nmeth.1314. 19305406

[51] M. Piecyk , J. Fauvre , C. Duret , C. Chaveroux , and C. Ferraro‐Peyret , “Surface SEnsing of Translation (SUnSET), a Method Based on Western Blot Assessing Protein Synthesis Rates in Vitro,” BIO‐PROTOCOL 14, no. 3 (2024): 4933, 10.21769/BioProtoc.4933. PMC10875356 38379826

[52] Y. Yang , Y. Zhu , S. Zhou , et al., “TRIM27 Cooperates With STK38L to Inhibit ULK1‐Mediated Autophagy and Promote Tumorigenesis,” The EMBO Journal 41, no. 14 (2022): 109777, 10.15252/embj.2021109777. PMC9289709 35670107

