# Supplementary material for: DCAF13 Safeguards Hematopoietic Stem Cells via RRS1‐Regulated Ribosome Biogenesis
Source: Adv Sci (Weinh). 2026 Mar 6;13(27):e13777. doi: 10.1002/advs.202513777 (PMC13170182; doi:10.1002/advs.202513777)
Supplement: Supplementary file 1 — Supporting File 1: advs74611‐sup‐0001‐SuppMat.docx. [file ADVS-13-e13777-s002.docx]

Supporting Information

**DCAF13 Safeguards Hematopoietic Stem Cells via RRS1-Regulated Ribosome Biogenesis**

**Contents:**

Supplemental Figure 1.

Supplemental Figure 2.

Supplemental Figure 3.

Supplemental Figure 4.

Supplemental Figure 5.

Supplemental Figure 6.

Supplemental Figure 7.

Supplemental Table 1-5 is provided and submitted as an individual file separately.


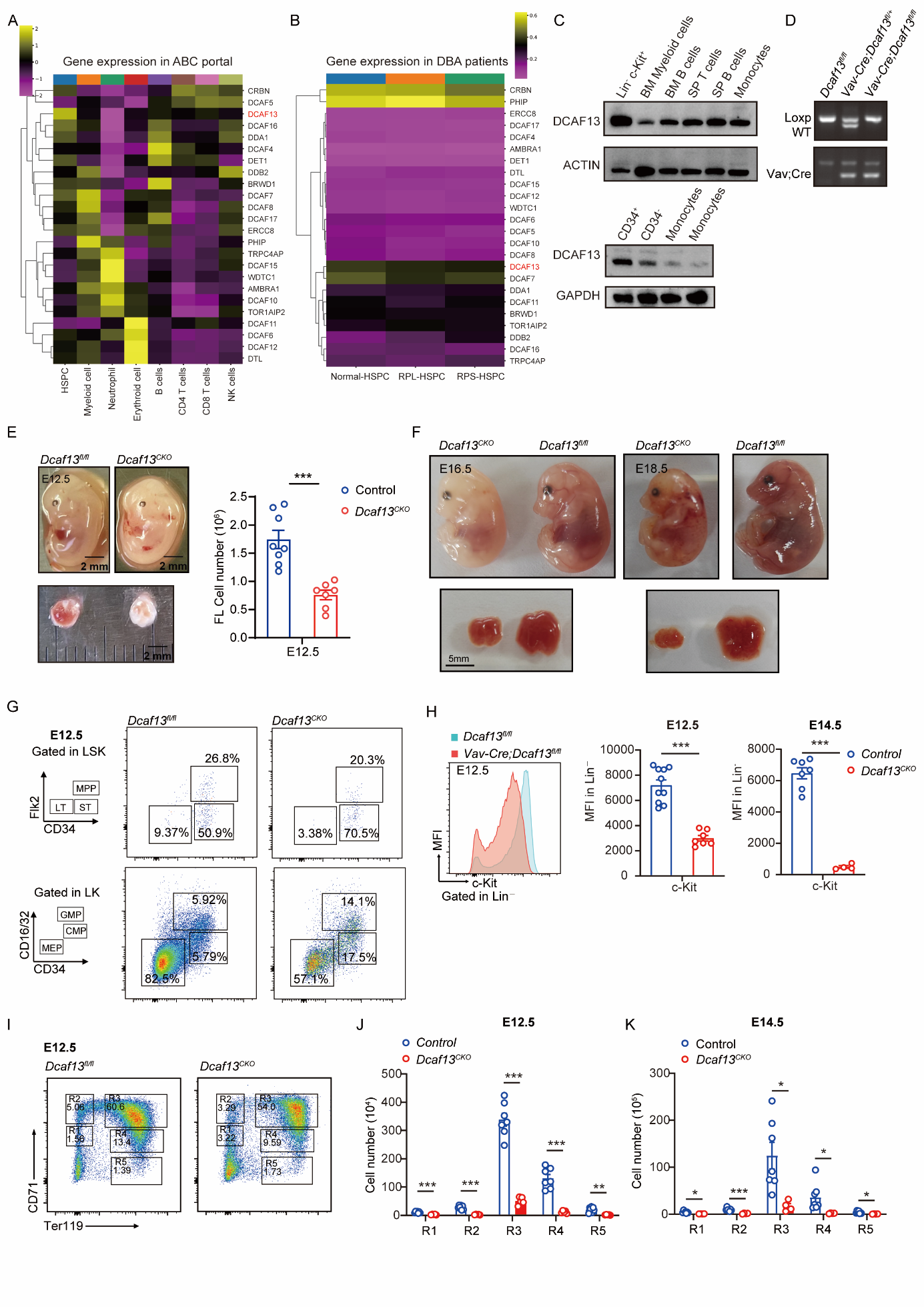


**Supplemental Figure 1.** ***Dcaf13* knockout embryos exhibit defective fetal liver hematopoiesis.**

**A** The expression level of DCAF13 in normal human blood cells (single-cell RNA-seq analysis obtained from ABC Portal: (http://abc.sklehabc.com)). **B** The expression level of DCAF13 in DBA (Diamond-Blackfan anemia) patients (single-cell RNA-seq data obtained from GSE156441 and GSE181830). **C** Western blot analysis of DCAF13 protein level in Lin^-^c-Kit^+^ cells, Myeloid cells, B cells and T cells isolated from mouse bone marrow and spleen. Human CD34^+^ cells were also enriched and subjected to Western blot analysis for DCAF13 protein level assessment. **D** Representative PCR to detect the *Dcaf13^loxP^* allele and *Vav-iCre*. **E** Left panel: representative image of *Dcaf13^fl/fl^*, *Dcaf13^CKO^* embryos and fetal livers at E12.5. Right panel: the cell numbers of E12.5 fetal livers. **F** Representative image of *Dcaf13^fl/fl^*, *Dcaf13^CKO^* embryos and fetal livers at E16.5 and E18.5. **G** The flow cytometry analysis of LT-HSC, ST-HSC, MPP, GMP, MEP and CMP in E12.5 fetal livers. **H** Histogram and quantification of MFI (Mean) values of c-Kit marker in E12.5 and E14.5 Lin^-^ cells; n=4-10. **I-K** Flow cytometry analysis of erythroid maturation in E12.5 and E14.5 fetal livers, including CD71^med^TER119^−^(R1), CD71^high^TER119^−^(R2), CD71^high^TER119^+^(R3), CD71^med^TER119^+^(R4), and CD71^low^TER119^+^(R5). Note that R1-R2 were immature erythroid cells, R3-R5 were mature erythroid cells; n=4-7. Data are presented as Mean ± SEM; * P < 0.05, ** P < 0.01, *** P < 0.001, Student’s t test.


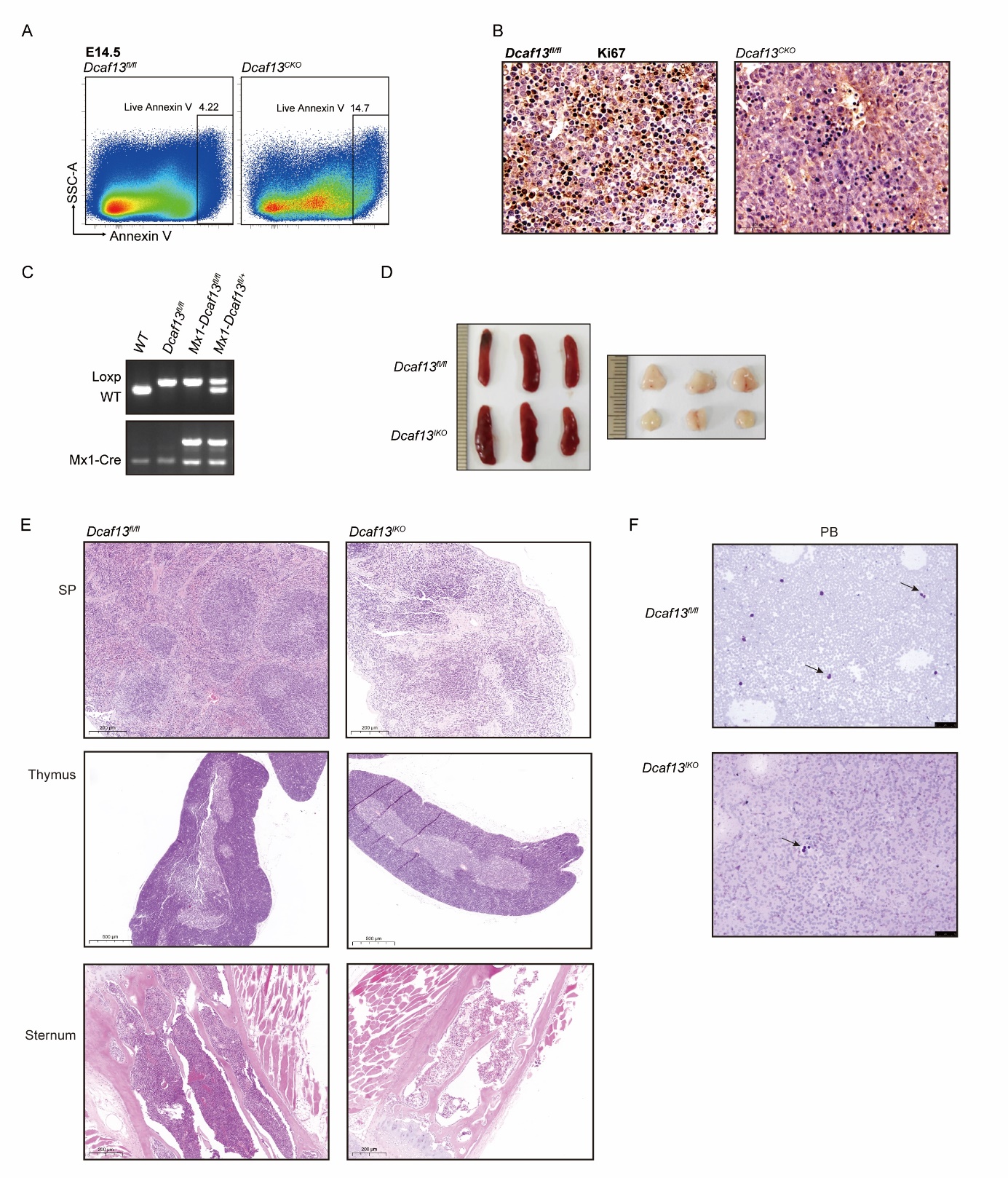


**Supplemental Figure 2. *Dcaf13* knockout results in morphological and histological abnormalities of hematopoietic organs in adult mice.**

**A** Representative FACS profiles of apoptosis in E14.5 fetal liver cells. **B** Ki67 staining of fetal liver tissues from *Dcaf13^fl/fl^* and *Dcaf13^CKO^* embryos at E14.5. **C** Representative PCR to detect the *Dcaf13^loxP^* allele and *Mx1-Cre*. **D** Representative images of spleen (SP) and thymus from *Dcaf13^fl/fl^* and *Dcaf13^fl/fl^;Mx1-Cre* mice after pIpC induction. **E** H&E staining of spleen, thymus and sternum from pIpC-treated *Dcaf13^fl/fl^* and *Dcaf13^fl/fl^;Mx1-Cre* mice. **F** Wright-Giemsa staining of the cytospin of peripheral blood (PB) from pIpC-treated *Dcaf13^fl/fl^* and *Dcaf13^fl/fl^;Mx1-Cre* mice. Data are presented as Mean ± SEM; * P < 0.05, ** P < 0.01, *** P < 0.001, Student’s t test.


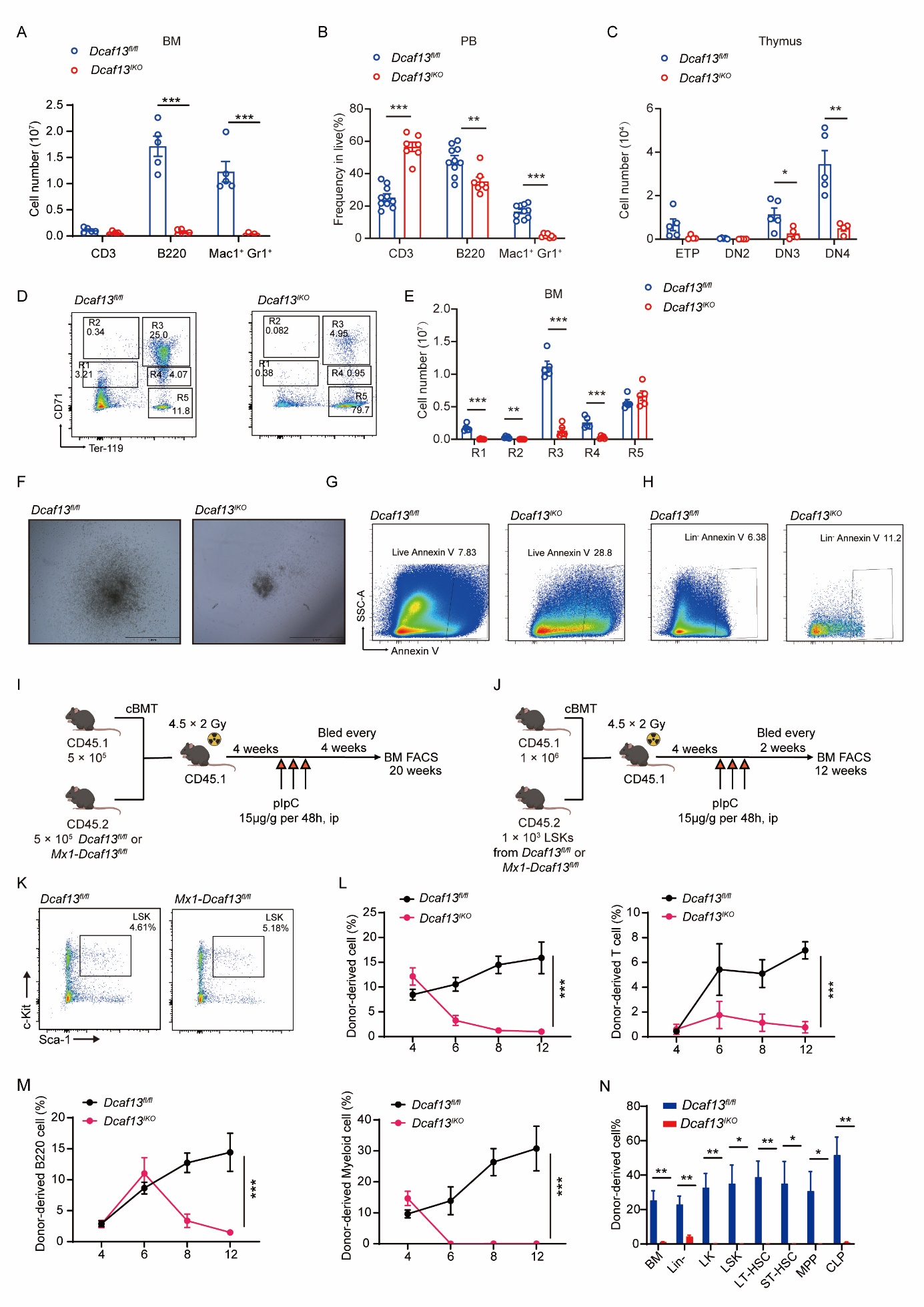


**Supplemental Figure 3. Depletion of *Dcaf13* results in hematopoietic failure in adult mice.**

**A** The cell numbers of T cells (CD3^+^), B cells (B220^+^) and myeloid cells (Mac1^+^Gr1^+^) in the BM of *Dcaf13^fl/fl^* and *Dcaf13^IKO^* mice; n=5. **B** The percentage of T cells, B cells and myeloid cells in the PB of *Dcaf13^fl/fl^* and *Dcaf13^IKO^* mice; n=7-10. **C** The cell number of progenitor T cells in the thymus from *Dcaf13^fl/fl^* and *Dcaf13^IKO^* mice; n=5. **D** Representative FACS analysis of erythroid maturation in the BM from *Dcaf13^fl/fl^* and *Dcaf13^IKO^* mice. **E** The absolute number of R1-R5 in the BM of *Dcaf13^fl/fl^* and *Dcaf13^IKO^* mice; n=5. **F** Representative images of granulocyte-macrophage colonies (GM) from *Dcaf13^fl/fl^* and *Dcaf13^IKO^* BM cells. **G-H** Apoptosis analysis of BM and Lin^-^ cells. **I** Strategy for competitive repopulation assay with *Dcaf13^fl/fl^* or *Dcaf13^fl/fl^*;*Mx1-Cre* mice. **J** Strategy for LSK competitive transplantation assay with *Dcaf13^fl/fl^* or *Dcaf13^fl/fl^*;*Mx1-Cre* mice. **K** Isolation of LSK cells. Flow cytometry plots demonstrating the gating strategy of LSKs from untreated *Dcaf13^fl/fl^* or *Dcaf13^fl/fl^*;*Mx1-Cre* mice. **L-M** Quantification of donor-derived (CD45.2^+^) cells in the PB of recipient mice at the indicated time points; n=7. **N** Donor contribution of indicated cell populations in BM of recipient mice 12 weeks post-transplantation; n=4. Data are presented as Mean ± SEM; * P < 0.05, ** P < 0.01, *** P < 0.001, Student’s t test.


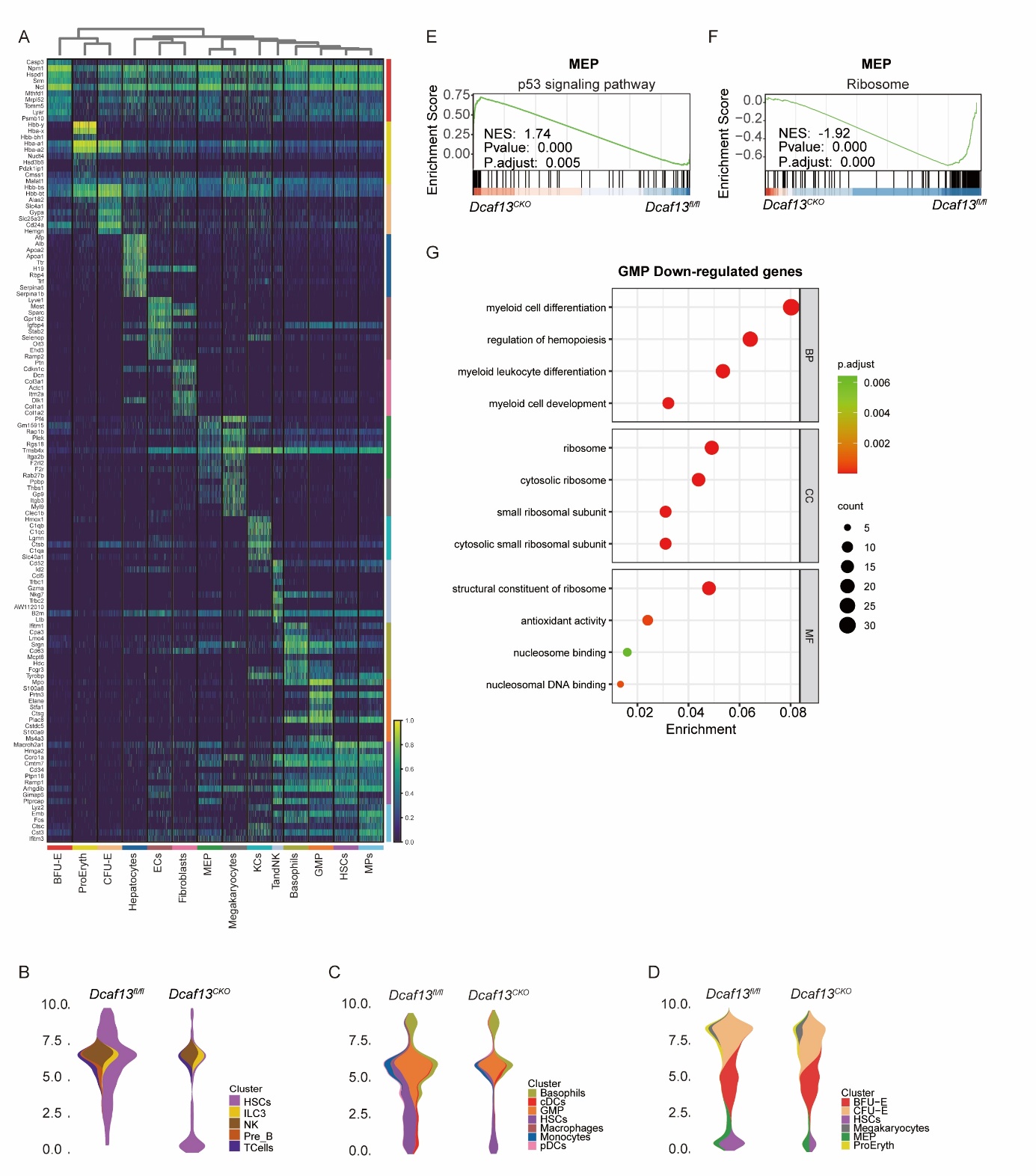


**Supplemental Figure 4. DCAF13 knockout impaired HSPC lineage commitment and disturbed P53 signaling pathway as well as ribosome biogenesis.**

**A** Heatmap of the top 10 expressed genes for each cluster identified by Seurat. **B-D** Single cell transcriptomes were divided into erythroid, lymphoid and myeloid differentiation trajectories using Monocle. The density plot showed the differentiation potential between *Dcaf13^fl/fl^* and *Dcaf13^CKO^* group. **E-F** GSEA analysis of the P53 signaling pathway and the ribosome in MEP cells between the *Dcaf13^fl/fl^* and the *Dcaf13^CKO^* group. **G** Enrichment analysis of down-regulated genes in *Dcaf13^CKO^* GMPs compared with *Dcaf13^fl/fl^* group.


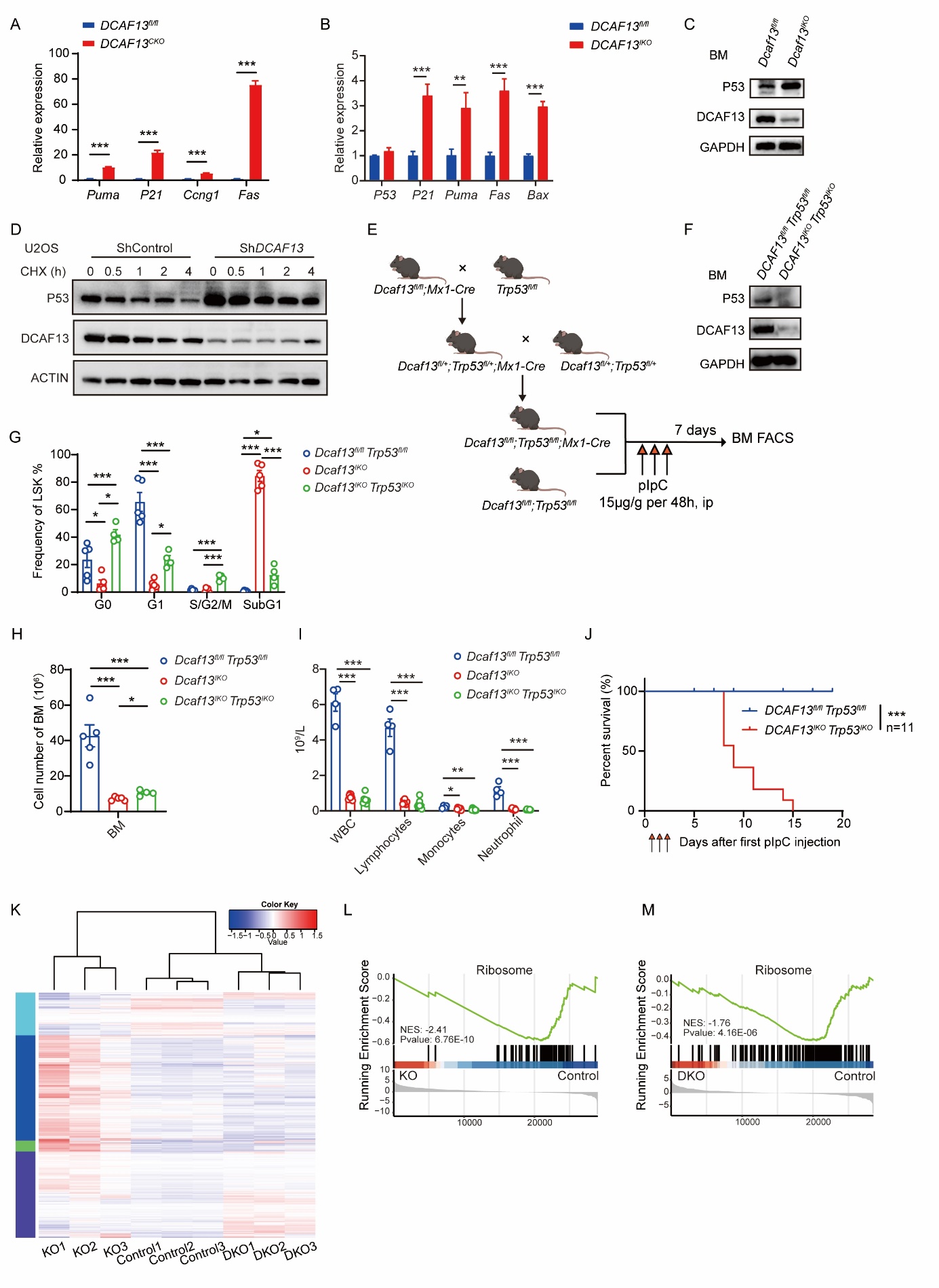


**Supplemental Figure 5**: **Double knockout of P53 and DCAF13 leads to acute lethal hematopoietic failure in adult mice.**

**A** The relative mRNA levels of p53 target genes between *Dcaf13^fl/fl^* and *Dcaf13^CKO^* Lin^-^ cells isolated from E14.5 fetal livers; n=3. **B** The mRNA expression levels of P53 and its target genes were quantified in *Dcaf13^IKO^* Lin^-^ cells; n=3. **C** Western blot analysis of P53 level in *Dcaf13^IKO^* BM cells. **D** p53 levels were determined in 100μg/ml cycloheximide (CHX)-treated DCAF13-KD U2OS cells for 0h, 0.5h, 1h, 2h and 4h. **E** The mating strategy used to obtain *Dcaf13^fl/fl^ Trp53^fl/fl^* and *Dcaf13^fl/fl^ Trp53^fl/fl^*;*Mx1-Cre* mice, which were then treated with pIpC to knock out *Dcaf13* and *Trp53*. **F** The protein level of P53 and DCAF13 in pIpC-treated *Dcaf13^fl/fl^ Trp53^fl/fl^* and *Dcaf13^IKO^ Trp53^IKO^* BM cells. **G** The frequencies of G0, G1 and S/G2/M phases in LSK cells were analyzed in *Dcaf13^fl/fl^ Trp53^fl/fl^*, *Dcaf13^IKO^* and *Dcaf13^IKO^ Trp53^IKO^* mice; n=4-5. **H** The cell number of BM in three groups of mice. **I** Complete blood count analysis of *Dcaf13^fl/fl^ Trp53^fl/fl^*, *Dcaf13^IKO^* and *Dcaf13^IKO^ Trp53^IKO^* mice; n=4. **J** Kaplan-Meier survival curve of mice following pIpC-induced *Dcaf13* and *Trp53* knockout; n=11. **K** Heatmap depicting global gene expression profiles across all three groups. **L-M** Gene set enrichment analysis (GSEA) of the ribosome biogenesis pathway in Lin⁻ cells, showing comparisons of *Dcaf13^IKO^* to *Dcaf13^fl/fl^* and *Dcaf13^IKO^ Trp53^IKO^* to *Dcaf13^fl/fl^* mice. Data are presented as Mean ± SEM. Comparisons among the three groups were analyzed by one-way ANOVA with Tukey’s post hoc test; * P < 0.05, ** P < 0.01, *** P < 0.001.


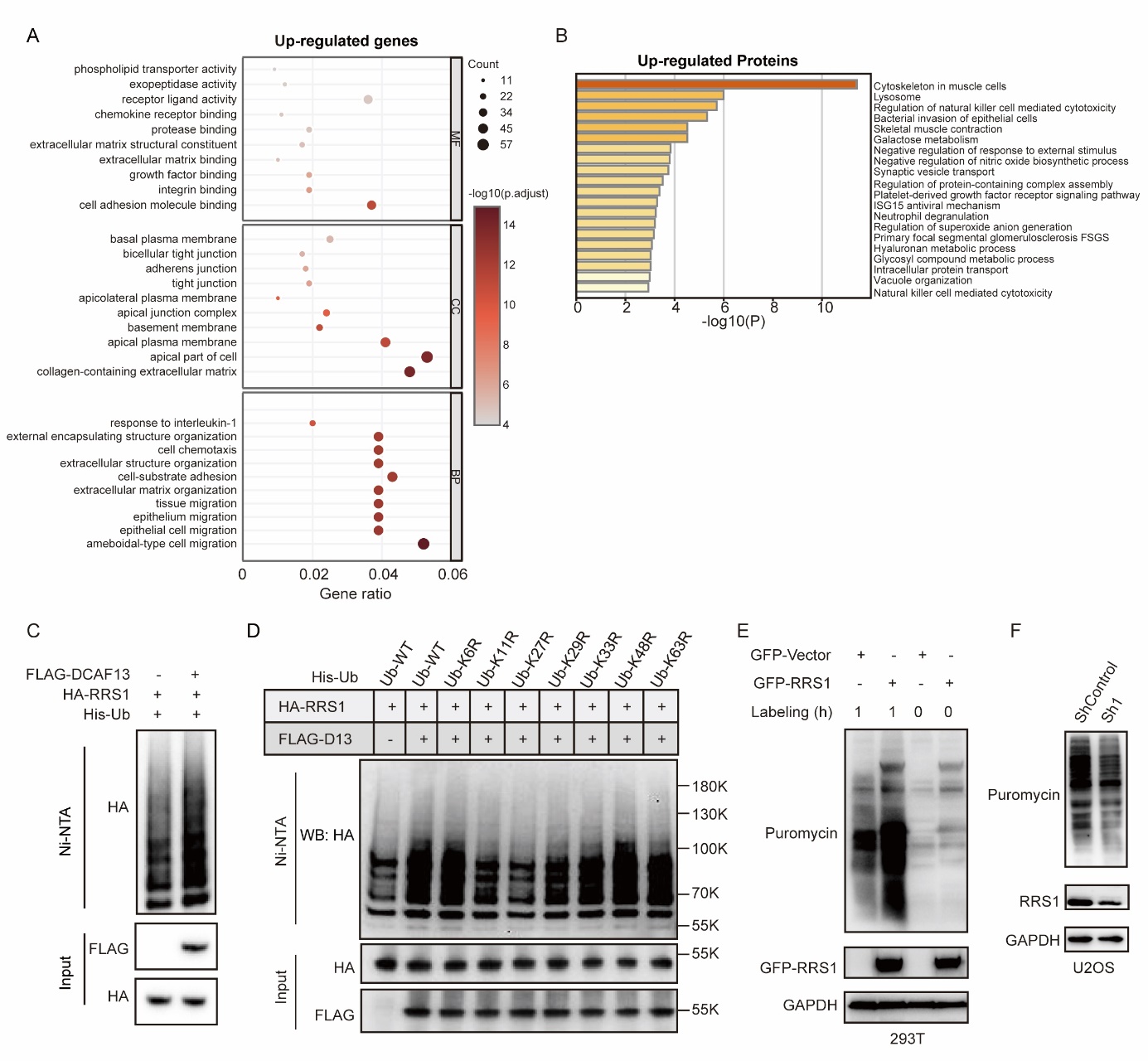


**Supplemental Figure 6. DCAF13 depletion impairs ribosome biogenesis, while RRS1 overexpression enhances protein synthesis.**

**A** GO enrichment pathway of Polysome-profiled up-regulated genes in *Dcaf13^CKO^* Lin^-^ cells compared with *Dcaf13^fl/fl^* controls. **B** Enrichment analysis of proteomics-identified up-regulated proteins in *Dcaf13^IKO^* Lin^-^ cells compared with *Dcaf13^fl/fl^* controls by Metascape (Zhou et al. Nature Commun. 2019 10(1):1523). **C** RRS1 ubiquitination assay in 293T cells transfected with HA-RRS1, FLAG-DCAF13 and His-ubiquitin for 48h. **D** Ubiquitination assay of HA‐RRS1 was measured in cells co‐expressing FLAG‐DCAF13 (FLAG-D13) with either His‐Ub WT, His‐Ub K6R, His‐Ub K11R, His‐Ub K27R, His‐Ub K29R, His‐Ub K33R, His‐Ub K48R and His‐Ub K63R respectively. **E** 293T cells were transfected with GFP-RRS1. 72h after transfection, cells were treated with 10μg/ml puromycin for 1 h. Puromycylation of nascent peptides was determined by Western blot. **F** *RRS1*-KD U2OS cells were treated with 10μg/ml puromycin for 1 h. Puromycylation of nascent peptides was determined by Western blot.


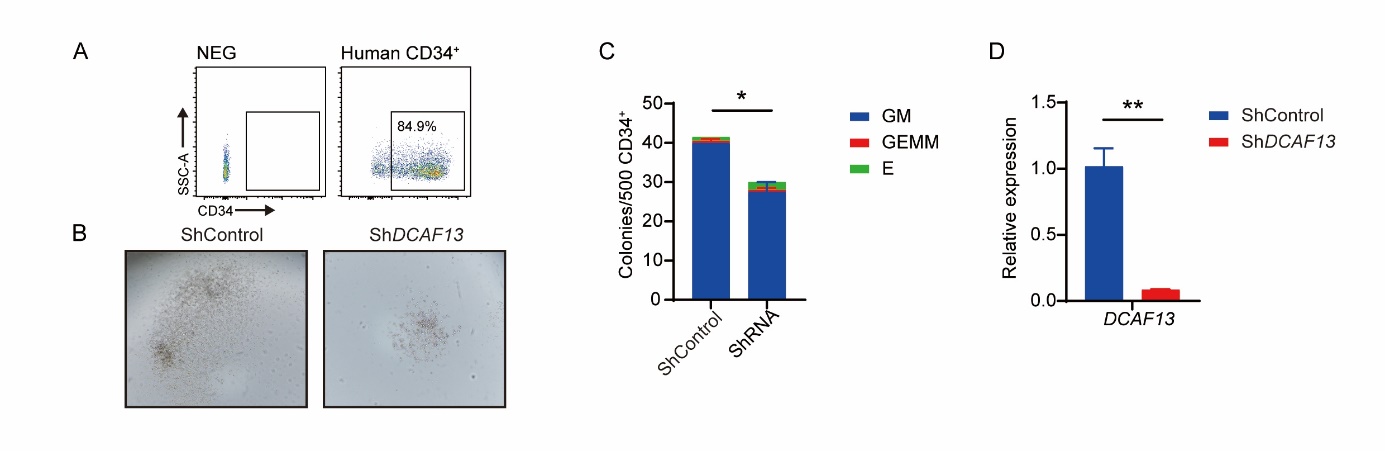


**Supplemental Figure 7. DCAF13 knockdown impairs the colony-forming capacity of human HSCs.**

**A** FACS analysis showing the proportion of CD34^+^ cells enriched from human umbilical cord blood. **B** Representative images of colonies formed by human CD34^+^ cells following 2-week culture in methylcellulose medium. **C** Quantification of colony numbers from human CD34⁺ cells after 2 weeks of culture. **D** The relative mRNA level of DCAF13 in human CD34^+^ cells, normalized to 18S; n=3. Data are presented as Mean ± SEM; * P < 0.05, ** P < 0.01, *** P < 0.001, Student’s t test.

**References:**

[1] S. H. Orkin, L. I. Zon, *Hematopoiesis: an evolving paradigm for stem cell biology*, *Cell* **2008**, *132* (4), 631-644, https://doi.org/10.1016/j.cell.2008.01.025.

[2] A. Mendelson, P. S. Frenette, *Hematopoietic stem cell niche maintenance during homeostasis and regeneration*, *Nat Med* **2014**, *20* (8), 833-846, https://doi.org/10.1038/nm.3647.

[3] S. Doulatov, F. Notta, E. Laurenti, J. E. Dick, *Hematopoiesis: a human perspective*, *Cell stem cell* **2012**, *10* (2), 120-136, https://doi.org/10.1016/j.stem.2012.01.006.

[4] R. A. Signer, J. A. Magee, A. Salic, S. J. Morrison, *Haematopoietic stem cells require a highly regulated protein synthesis rate*, *Nature* **2014**, *509* (7498), 49-54, https://doi.org/10.1038/nature13035.

[5] R. K. Khajuria, M. Munschauer, J. C. Ulirsch, C. Fiorini, L. S. Ludwig, S. K. McFarland, N. J. Abdulhay, H. Specht, H. Keshishian, D. R. Mani, M. Jovanovic, S. R. Ellis, C. P. Fulco, J. M. Engreitz, S. Schütz, J. Lian, K. W. Gripp, O. K. Weinberg, G. S. Pinkus, L. Gehrke, A. Regev, E. S. Lander, H. T. Gazda, W. Y. Lee, V. G. Panse, S. A. Carr, V. G. Sankaran, *Ribosome Levels Selectively Regulate Translation and Lineage Commitment in Human Hematopoiesis*, *Cell* **2018**, *173* (1), 90-103.e119, https://doi.org/10.1016/j.cell.2018.02.036.

[6] H. Wang, Z. Zhang, C. Han, P. Jiang, J. Xu, Y. Han, D. Huang, J. Li, J. Zhou, M. Durnin, S. Chen, Y. Liu, J. Sheng, J. Cao, J. Liu, B. Liu, J. Yu, F. Wang, P. Qian, *SNORD113-114 cluster maintains haematopoietic stem cell self-renewal via orchestrating the translation machinery*, *Nat Cell Biol* **2025**, *27* (2), 246-261, https://doi.org/10.1038/s41556-024-01593-7.

[7] Z. Zheng, S. Yang, F. Gou, C. Tang, Z. Zhang, Q. Gu, G. Sun, P. Jiang, N. Wang, X. Zhao, J. Kang, Y. Wang, Y. He, M. Yang, T. Lu, S. Lu, P. Qian, P. Zhu, H. Cheng, T. Cheng, *The ATF4-RPS19BP1 axis modulates ribosome biogenesis to promote erythropoiesis*, *Blood* **2024**, *144* (7), 742-756, https://doi.org/10.1182/blood.2023021901.

[8] A. J. Warren, *Molecular basis of the human ribosomopathy Shwachman-Diamond syndrome*, *Adv Biol Regul* **2018**, *67*, 109-127, https://doi.org/10.1016/j.jbior.2017.09.002.

[9] C. R. Reilly, A. Shimamura, *Predisposition to myeloid malignancies in Shwachman-Diamond syndrome: biological insights and clinical advances*, *Blood* **2023**, *141* (13), 1513-1523, https://doi.org/10.1182/blood.2022017739.

[10] L. Da Costa, T. Leblanc, N. Mohandas, *Diamond-Blackfan anemia*, *Blood* **2020**, *136* (11), 1262-1273, https://doi.org/10.1182/blood.2019000947.

[11] N. Kawashima, U. Oyarbide, M. Cipolli, V. Bezzerri, S. J. Corey, *Shwachman-Diamond syndromes: clinical, genetic, and biochemical insights from the rare variants*, *Haematologica* **2023**, *108* (10), 2594-2605, https://doi.org/10.3324/haematol.2023.282949.

[12] J. Zhang, Y. L. Zhang, L. W. Zhao, J. X. Guo, J. L. Yu, S. Y. Ji, L. R. Cao, S. Y. Zhang, L. Shen, X. H. Ou, H. Y. Fan, *Mammalian nucleolar protein DCAF13 is essential for ovarian follicle maintenance and oocyte growth by mediating rRNA processing*, *Cell Death Differ* **2019**, *26* (7), 1251-1266, https://doi.org/10.1038/s41418-018-0203-7.

[13] J. Lee, P. Zhou, *DCAFs, the missing link of the CUL4-DDB1 ubiquitin ligase*, *Mol Cell* **2007**, *26* (6), 775-780, https://doi.org/10.1016/j.molcel.2007.06.001.

[14] S. Jackson, Y. Xiong, *CRL4s: the CUL4-RING E3 ubiquitin ligases*, *Trends Biochem Sci* **2009**, *34* (11), 562-570, https://doi.org/10.1016/j.tibs.2009.07.002.

[15] S. Angers, T. Li, X. Yi, M. J. MacCoss, R. T. Moon, N. Zheng, *Molecular architecture and assembly of the DDB1-CUL4A ubiquitin ligase machinery*, *Nature* **2006**, *443* (7111), 590-593, https://doi.org/10.1038/nature05175.

[16] S. Boulon, B. J. Westman, S. Hutten, F. M. Boisvert, A. I. Lamond, *The nucleolus under stress*, *Molecular cell* **2010**, *40* (2), 216-227, https://doi.org/10.1016/j.molcel.2010.09.024.

[17] M. C. Lafita-Navarro, M. Conacci-Sorrell, *Nucleolar stress: From development to cancer*, *Semin Cell Dev Biol* **2023**, *136*, 64-74, https://doi.org/10.1016/j.semcdb.2022.04.001.

[18] M. Dumble, L. Moore, S. M. Chambers, H. Geiger, G. Van Zant, M. A. Goodell, L. A. Donehower, *The impact of altered p53 dosage on hematopoietic stem cell dynamics during aging*, *Blood* **2007**, *109* (4), 1736-1742, https://doi.org/10.1182/blood-2006-03-010413.

[19] Y. Liu, S. E. Elf, Y. Miyata, G. Sashida, Y. Liu, G. Huang, S. Di Giandomenico, J. M. Lee, A. Deblasio, S. Menendez, J. Antipin, B. Reva, A. Koff, S. D. Nimer, *p53 regulates hematopoietic stem cell quiescence*, *Cell stem cell* **2009**, *4* (1), 37-48, https://doi.org/10.1016/j.stem.2008.11.006.

[20] A. James, Y. Wang, H. Raje, R. Rosby, P. DiMario, *Nucleolar stress with and without p53*, *Nucleus* **2014**, *5* (5), 402-426, https://doi.org/10.4161/nucl.32235.

[21] T. Teng, G. Thomas, C. A. Mercer, *Growth control and ribosomopathies*, *Curr Opin Genet Dev* **2013**, *23* (1), 63-71, https://doi.org/10.1016/j.gde.2013.02.001.

[22] N. J. Boon, R. A. Oliveira, P. R. Körner, A. Kochavi, S. Mertens, Y. Malka, R. Voogd, S. E. M. van der Horst, M. A. Huismans, L. P. Smabers, J. M. Draper, L. F. A. Wessels, P. Haahr, J. M. L. Roodhart, T. N. M. Schumacher, H. J. Snippert, R. Agami, T. R. Brummelkamp, *DNA damage induces p53-independent apoptosis through ribosome stalling*, *Science* **2024**, *384* (6697), 785-792, https://doi.org/10.1126/science.adh7950.

[23] A. Stedman, S. Beck-Cormier, M. Le Bouteiller, A. Raveux, S. Vandormael-Pournin, S. Coqueran, V. Lejour, L. Jarzebowski, F. Toledo, S. Robine, M. Cohen-Tannoudji, *Ribosome biogenesis dysfunction leads to p53-mediated apoptosis and goblet cell differentiation of mouse intestinal stem/progenitor cells*, *Cell Death Differ* **2015**, *22* (11), 1865-1876, https://doi.org/10.1038/cdd.2015.57.

[24] A. Ogawa, K. Izumikawa, S. Tate, S. Isoyama, M. Mori, K. Fujiwara, S. Watanabe, T. Ohga, U. Jo, D. Taniyama, S. Kitajima, S. Tanaka, H. Onji, S. I. Kageyama, G. Yamamoto, H. Saito, T. Y. Morita, M. Okada, M. Natsumeda, M. Nagahama, J. Kobayashi, A. Ohashi, H. Sasanuma, S. Higashiyama, S. Dan, Y. Pommier, J. Murai, *SLFN11-mediated ribosome biogenesis impairment induces TP53-independent apoptosis*, *Molecular cell* **2025**, *85* (5), 894-912.e810, https://doi.org/10.1016/j.molcel.2025.01.008.

[25] Y. L. Zhang, L. W. Zhao, J. Zhang, R. Le, S. Y. Ji, C. Chen, Y. Gao, D. Li, S. Gao, H. Y. Fan, *DCAF13 promotes pluripotency by negatively regulating SUV39H1 stability during early embryonic development*, *The EMBO journal* **2018**, *37* (18), https://doi.org/10.15252/embj.201898981.

[26] Q. Zhou, X. Li, N. Wang, L. Zhang, E. Jiang, K. Wang, X. Yan, C. Zhang, *DCAF13 is essential for mouse uterine function and fertility*, *Cell Death Discov* **2025**, *11* (1), 359, https://doi.org/10.1038/s41420-025-02583-w.

[27] X. Gao, F. Hong, Z. Hu, Z. Zhang, Y. Lei, X. Li, T. Cheng, *ABC portal: a single-cell database and web server for blood cells*, *Nucleic acids research* **2023**, *51* (D1), D792-d804, https://doi.org/10.1093/nar/gkac646.

[28] T. Yokomizo, T. Ideue, S. Morino-Koga, C. Y. Tham, T. Sato, N. Takeda, Y. Kubota, M. Kurokawa, N. Komatsu, M. Ogawa, K. Araki, M. Osato, T. Suda, *Independent origins of fetal liver haematopoietic stem and progenitor cells*, *Nature* **2022**, *609* (7928), 779-784, https://doi.org/10.1038/s41586-022-05203-0.

[29] M. H. Baron, J. Isern, S. T. Fraser, *The embryonic origins of erythropoiesis in mammals*, *Blood* **2012**, *119* (21), 4828-4837, https://doi.org/10.1182/blood-2012-01-153486.

[30] W. Wei, X. Gao, J. Qian, L. Li, C. Zhao, L. Xu, Y. Zhu, Z. Liu, N. Liu, X. Wang, Z. Jin, B. Liu, L. Xu, J. Dong, S. Zhang, J. Wang, Y. Zhang, Y. Yu, Z. Yan, Y. Yang, J. Lu, Y. Fang, N. Yuan, J. Wang, *Beclin 1 prevents ISG15-mediated cytokine storms to secure fetal hematopoiesis and survival*, *J Clin Invest* **2025**, *135* (3), https://doi.org/10.1172/jci177375.

[31] S. Wei, J. Xing, J. Chen, L. Chen, J. Lv, X. Chen, T. Li, T. Yu, H. Wang, K. Wang, W. Yu, *DCAF13 inhibits the p53 signaling pathway by promoting p53 ubiquitination modification in lung adenocarcinoma*, *J Exp Clin Cancer Res* **2024**, *43* (1), 3, https://doi.org/10.1186/s13046-023-02936-2.

[32] A. P. Schuller, R. Green, *Roadblocks and resolutions in eukaryotic translation*, *Nat Rev Mol Cell Biol* **2018**, *19* (8), 526-541, https://doi.org/10.1038/s41580-018-0011-4.

[33] K. Dörner, C. Ruggeri, I. Zemp, U. Kutay, *Ribosome biogenesis factors-from names to functions*, *The EMBO journal* **2023**, *42* (7), e112699, https://doi.org/10.15252/embj.2022112699.

[34] S. Kharde, F. R. Calviño, A. Gumiero, K. Wild, I. Sinning, *The structure of Rpf2-Rrs1 explains its role in ribosome biogenesis*, *Nucleic acids research* **2015**, *43* (14), 7083-7095, https://doi.org/10.1093/nar/gkv640.

[35] N. Asano, K. Kato, A. Nakamura, K. Komoda, I. Tanaka, M. Yao, *Structural and functional analysis of the Rpf2-Rrs1 complex in ribosome biogenesis*, *Nucleic acids research* **2015**, *43* (9), 4746-4757, https://doi.org/10.1093/nar/gkv305.

[36] P. Burda, P. Laslo, T. Stopka, *The role of PU.1 and GATA-1 transcription factors during normal and leukemogenic hematopoiesis*, *Leukemia* **2010**, *24* (7), 1249-1257, https://doi.org/10.1038/leu.2010.104.

[37] H. Iwasaki, C. Somoza, H. Shigematsu, E. A. Duprez, J. Iwasaki-Arai, S. Mizuno, Y. Arinobu, K. Geary, P. Zhang, T. Dayaram, M. L. Fenyus, S. Elf, S. Chan, P. Kastner, C. S. Huettner, R. Murray, D. G. Tenen, K. Akashi, *Distinctive and indispensable roles of PU.1 in maintenance of hematopoietic stem cells and their differentiation*, *Blood* **2005**, *106* (5), 1590-1600, https://doi.org/10.1182/blood-2005-03-0860.

[38] J. E. Kim, X. Pan, K. Y. Tse, H. H. Chan, C. Dong, M. S. Y. Huen, *PHF8 facilitates transcription recovery following DNA double-strand break repair*, *Nucleic acids research* **2024**, *52* (17), 10297-10310, https://doi.org/10.1093/nar/gkae661.

[39] Z. Z. Yang, B. Yang, H. Yan, X. Ma, B. Tian, B. Zheng, Y. X. Chen, Y. M. Dong, J. Deng, Z. Zhan, Y. Shi, J. Y. Zhang, D. Lu, J. H. He, Y. Zhang, K. Hu, S. Zhu, K. Zhou, Y. C. Zhang, Y. Zheng, D. Yin, J. Y. Liao, *DCAF13-mediated K63-linked ubiquitination of RNA polymerase I promotes uncontrolled proliferation in Breast Cancer*, *Nat Commun* **2025**, *16* (1), 557, https://doi.org/10.1038/s41467-025-55851-9.

[40] S. Chen, J. Lin, Z. Yang, Y. Wang, Q. Wang, D. Wang, Y. Qu, Q. Lin, J. Liu, S. Yan, Z. Wang, X. Qian, Y. Xiao, X. Li, Y. Chen, W. Fang, J. Zhao, Z. Lu, H. Ren, Y. Zhu, L. Ma, *TRIM24-mediated K27-linked ubiquitination of ULK1 alleviates energy stress-induced autophagy and promote prostate cancer growth in the context of SPOP mutation*, *Cell Death Differ* **2025**, https://doi.org/10.1038/s41418-025-01582-9.

[41] H. Rho, S. Kim, S. U. Kim, J. W. Kim, S. H. Lee, S. H. Park, F. E. Escorcia, J. Y. Chung, J. Song, *CHIP ameliorates nonalcoholic fatty liver disease via promoting K63- and K27-linked STX17 ubiquitination to facilitate autophagosome-lysosome fusion*, *Nat Commun* **2024**, *15* (1), 8519, https://doi.org/10.1038/s41467-024-53002-0.

[42] D. Zhao, G. Zhong, J. Li, J. Pan, Y. Zhao, H. Song, W. Sun, X. Jin, Y. Li, R. Du, J. Nie, T. Liu, J. Zheng, Y. Jia, Z. Liu, W. Liu, X. Yuan, Z. Liu, J. Song, G. Kan, Y. Li, C. Liu, X. Gao, W. Xing, Y. Z. Chang, Y. Li, S. Ling, *Targeting E3 Ubiquitin Ligase WWP1 Prevents Cardiac Hypertrophy Through Destabilizing DVL2 via Inhibition of K27-Linked Ubiquitination*, *Circulation* **2021**, *144* (9), 694-711, https://doi.org/10.1161/circulationaha.121.054827.

[43] J. Zhang, P. Harnpicharnchai, J. Jakovljevic, L. Tang, Y. Guo, M. Oeffinger, M. P. Rout, S. L. Hiley, T. Hughes, J. L. Woolford, Jr., *Assembly factors Rpf2 and Rrs1 recruit 5S rRNA and ribosomal proteins rpL5 and rpL11 into nascent ribosomes*, *Genes Dev* **2007**, *21* (20), 2580-2592, https://doi.org/10.1101/gad.1569307.

[44] P. Cao, A. Yang, P. Li, X. Xia, Y. Han, G. Zhou, R. Wang, F. Yang, Y. Li, Y. Zhang, Y. Cui, H. Ji, L. Lu, F. He, G. Zhou, *Genomic gain of RRS1 promotes hepatocellular carcinoma through reducing the RPL11-MDM2-p53 signaling*, *Sci Adv* **2021**, *7* (35), https://doi.org/10.1126/sciadv.abf4304.

[45] A. C. Vind, A. V. Genzor, S. Bekker-Jensen, *Ribosomal stress-surveillance: three pathways is a magic number*, *Nucleic acids research* **2020**, *48* (19), 10648-10661, https://doi.org/10.1093/nar/gkaa757.

[46] L. Zhou, S. Wang, W. Hu, X. Liu, L. Xu, B. Tong, T. Zhang, Z. Xue, Y. Guo, J. Zhao, L. Lu, H. Fan, W. Qian, J. Chen, W. Chen, L. Wang, *T cell proliferation requires ribosomal maturation in nucleolar condensates dependent on DCAF13*, *The Journal of cell biology* **2023**, *222* (10), https://doi.org/10.1083/jcb.202201096.

[47] E. W. Mills, R. Green, *Ribosomopathies: There's strength in numbers*, *Science* **2017**, *358* (6363), https://doi.org/10.1126/science.aan2755.

[48] A. Narla, B. L. Ebert, *Ribosomopathies: human disorders of ribosome dysfunction*, *Blood* **2010**, *115* (16), 3196-3205, https://doi.org/10.1182/blood-2009-10-178129.

[49] M. Li, C. Qiu, Y. Bian, D. Shi, B. Wang, Q. Ma, X. Wang, J. Shi, L. Zhang, Y. Ma, P. Zhu, T. Cheng, Y. Chu, W. Yuan, *SETD5 modulates homeostasis of hematopoietic stem cells by mediating RNA Polymerase II pausing in cooperation with HCF-1*, *Leukemia* **2022**, *36* (4), 1111-1122, https://doi.org/10.1038/s41375-021-01481-1.

[50] E. K. Schmidt, G. Clavarino, M. Ceppi, P. Pierre, *SUnSET, a nonradioactive method to monitor protein synthesis*, *Nat Methods* **2009**, *6* (4), 275-277, https://doi.org/10.1038/nmeth.1314.

[51] M. Piecyk, J. Fauvre, C. Duret, C. Chaveroux, C. Ferraro-Peyret, *SUrface SEnsing of Translation (SUnSET), a Method Based on Western Blot Assessing Protein Synthesis Rates in vitro*, *Bio Protoc* **2024**, *14* (3), e4933, https://doi.org/10.21769/BioProtoc.4933.

[52] Y. Yang, Y. Zhu, S. Zhou, P. Tang, R. Xu, Y. Zhang, D. Wei, J. Wen, R. F. Thorne, X. D. Zhang, J. L. Guan, L. Liu, M. Wu, S. Chen, *TRIM27 cooperates with STK38L to inhibit ULK1-mediated autophagy and promote tumorigenesis*, *The EMBO journal* **2022**, *41* (14), e109777, https://doi.org/10.15252/embj.2021109777.
